# Viscoelasticity and advective flow of RNA underlies nucleolar form and function

**DOI:** 10.1016/j.molcel.2023.08.006

**Published:** 2023-09-07

**Authors:** Joshua A. Riback, Jorine M. Eeftens, Daniel S.W. Lee, Sofia A. Quinodoz, Anita Donlic, Natalia Orlovsky, Lennard Wiesner, Lien Beckers, Lindsay A. Becker, Amy R. Strom, Ushnish Rana, Michele Tolbert, Byron W. Purse, Ralph Kleiner, Richard Kriwacki, Clifford P. Brangwynne

**Affiliations:** 1 Department of Chemical and Biological Engineering, Princeton University, Princeton, NJ 08544, USA; 2 Lewis-Sigler Institute for Integrative Genomics, Princeton University, Princeton, Princeton, NJ 08544, USA; 3 Princeton Institute for the Science and Technology of Materials, Princeton University, Princeton, Princeton, NJ 08544, USA; 4 Howard Hughes Medical Institute, Princeton University, Princeton, Princeton, NJ 08544, USA; 5 Department of Molecular Biology, Princeton University, Princeton, Princeton, NJ 08544, USA; 6 Department of Structural Biology, St. Jude Children’s Research Hospital, Memphis, TN 38103, USA; 7 Department of Chemistry and Biochemistry and the Viral Information Institute, San Diego State University, San Diego, CA 92182, USA; 8 Department of Chemistry, Princeton University, Princeton, Princeton, NJ 08544, USA; 9 Omenn-Darling Bioengineering Institute, Princeton University, Princeton, NJ 08544, USA; 10 These authors contributed equally; 11 Present address: Department of Molecular and Cellular Biology, Baylor College of Medicine, Houston, TX 77030, USA; 12 Present address: Department of Cell Biology, Radboud University, Faculty of Science, Radboud Institute for Molecular Life Sciences, Nijmegen; 13 Present address: Biological and Biomedical Sciences Program, Harvard University; 14 Lead contact

## Abstract

The nucleolus is the largest biomolecular condensate and facilitates transcription, processing, and assembly of ribosomal RNA (rRNA). Although nucleolar function is thought to require multiphase liquid-like properties, nucleolar fluidity and its connection to the highly coordinated transport and biogenesis of ribosomal subunits are poorly understood. Here, we use quantitative imaging, mathematical modeling, and pulse-chase nucleotide labeling to examine nucleolar material properties and rRNA dynamics. The mobility of rRNA is several orders of magnitude slower than that of nucleolar proteins, with rRNA steadily moving away from the transcriptional sites in a slow (~1 Å/s), radially directed fashion. This constrained but directional mobility, together with polymer physics-based calculations, suggests that nascent rRNA forms an entangled gel, whose constant production drives outward flow. We propose a model in which progressive maturation of nascent rRNA reduces its initial entanglement, fluidizing the nucleolar periphery to facilitate the release of assembled pre-ribosomal particles.

## In brief

Riback et al. use quantitative methods to investigate nucleolar material state and ribosomal RNA transport. They find that nucleoli are not simple liquids but, in fact, complex fluids, providing new insights into the role of the nucleolus in ribosome biogenesis.

## Graphical abstract

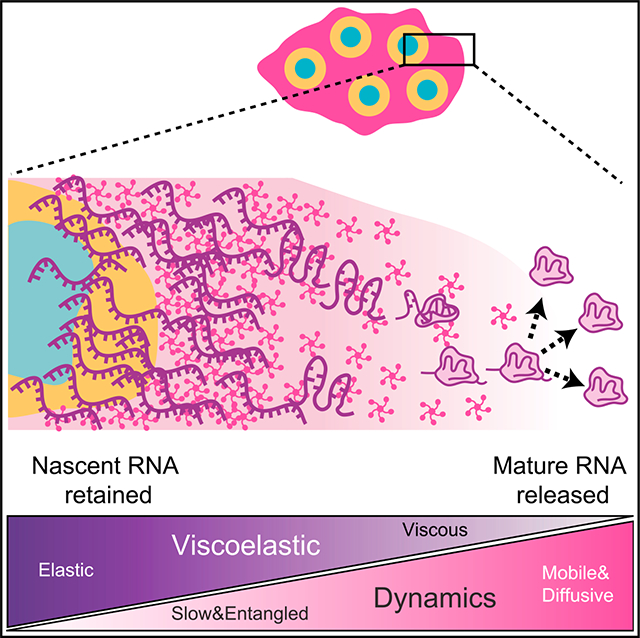

## INTRODUCTION

Cells compartmentalize biomolecules into organelles to enable spatiotemporal control over the formation, processing, and regulation of the macromolecular complexes that are essential for life. An important class of such compartments is biomolecular condensates, which, despite their lack of a bounding membrane, concentrate biomolecules into often liquid-like organelles with roughly spherical shapes, some degree of internal mixing, and exchange with the surroundings.^1–3^ These dynamic features are facilitated through weak multivalent interactions, typically involving oligomerized intrinsically disordered regions (IDRs), which drive phase separation and related phase transitions^4^ and provide internal cohesivity to the constantly restructuring biomolecular network.^5–8^

Despite a plethora of studies supporting the phase-separation concept, the exact physical nature of biomolecular condensates has been debated.^9,10^ This sometimes arises from over-simplified representations, including the binary characterization of condensates as either purely liquid-like, or else reflecting some qualitatively different assembly process. Such simplifications ignore the inherent biological complexities of intracellular condensates, including their polymeric nature,^11,12^ compositional complexity,^13^ and the role of active processes.^14^ For example, condensates typically contain hundreds of different components, which contribute to the thermodynamic driving forces of phase separation in ways that are not captured with two-component mean-field models.^15–17^ A full picture of how condensates contribute to biological function will require an understanding of how they differ from simple liquids and how those properties are exploited by cells to impart biological function.^18^

One of the most essential cellular processes occurs in the nucleolus, the most prominent condensate, which is the site of ribosomal subunit assembly in all eukaryotes.^19^ This process was first visualized in “Christmas tree”-like electron microscopy spreads of isolated amphibian nucleoli, in which a “tree trunk” containing ribosomal DNA (rDNA) repeats extends tightly packed “branches” of nascent ribosomal RNA (rRNA) transcripts, followed by terminal “balls” containing early rRNA processing machinery.^20,21^ In human nucleoli, this process is organized in three distinct and concentrically ordered layers, the fibrillar center (FC), dense fibrillar component (DFC), and granular component (GC), which roughly correspond to the sites of rRNA transcription, processing, and assembly into the small (pre-40S) and large (pre-60S) ribosomal subunits (RSUs), respectively.^22^ Corresponding to these steps, the three layers harbor components necessary for each process—the FCs contain RNA polymerase I subunits and transcription factors and form at rDNA repeat-containing genes in nucleolar organizer regions (NORs).^23^ As nascent rRNA is transcribed, enzymes such as fibrillarin (FBL, part of the C/D Box snoRNP complex) localize to the DFC to promote initial rRNA modification and cleavage steps.^24,25^ Finally, the GC contains various proteins that assist in further rRNA processing and binding to ribosomal proteins (r-proteins), ultimately forming nearly complete RSUs, which then move into the nucleoplasm and the cytoplasm.^22,26,27^ The size of the compartments suggests that rRNA processing and folding in the GC are the ratelimiting steps for RSU assembly.

The distinct nucleolar layers are thought to represent immiscible liquid-like subphases, which each form via phase separation but do not mix, instead remaining nested due to their “apparent” differences in surface tension.^25,28–30^ These immiscible phases have been suggested to facilitate the sequential processing of rRNA transcripts, possibly through a “hand-off” mechanism, whereby sequential processing steps occur in an assembly line.^29,31^ The transfer of rRNA into and out of the GC is linked to the thermodynamics that drives its phase separation—the effective interaction valency of rRNA decreases as it is assembled into ribosomal subunits, resulting in their eventual thermodynamic expulsion.^15,27^

Despite the compelling simplicity of this model, it cannot alone account for the orderly and well-coordinated hand-off of rRNA and associated proteins, involving the dozens of different processing steps that have been characterized with extensive genetic and biochemical studies^32,33^ and, more recently, with advanced structural and bioinformatics studies.^26,27,34^ Indeed, if the nucleolus is a liquid, even one comprising several distinct liquid layers, then free diffusion within each layer could result in non-sequential and aberrant rRNA modification, processing, and binding to associated proteins. Thus, it is unclear how to reconcile the ordered nature of ribosome biogenesis with the apparent fluidity of the nucleolus as a liquid-phase reaction crucible.

In this manuscript, we show that nucleolar form and function are closely linked to the constrained mobility of rRNA, which exhibits slow, steady, and substantially directional flow as it moves toward the nucleolar periphery. Experiments and modeling support a physical picture in which nascent rRNA is highly entangled, exhibiting behavior consistent with a partially solid-like viscoelastic gel that is steadily pushed outward through the GC. Our findings suggest that nucleolar proteins may facilitate ribosome biogenesis in part by modulating the degree of rRNA entanglement, with fully processed pre-ribosomal particles released from these constraints and partitioned into the nucleoplasm from the more liquid-like nucleolar periphery.

## RESULTS

### Nucleoli exhibit rapid protein exchange but nonspherical morphology

**Figure 1.**
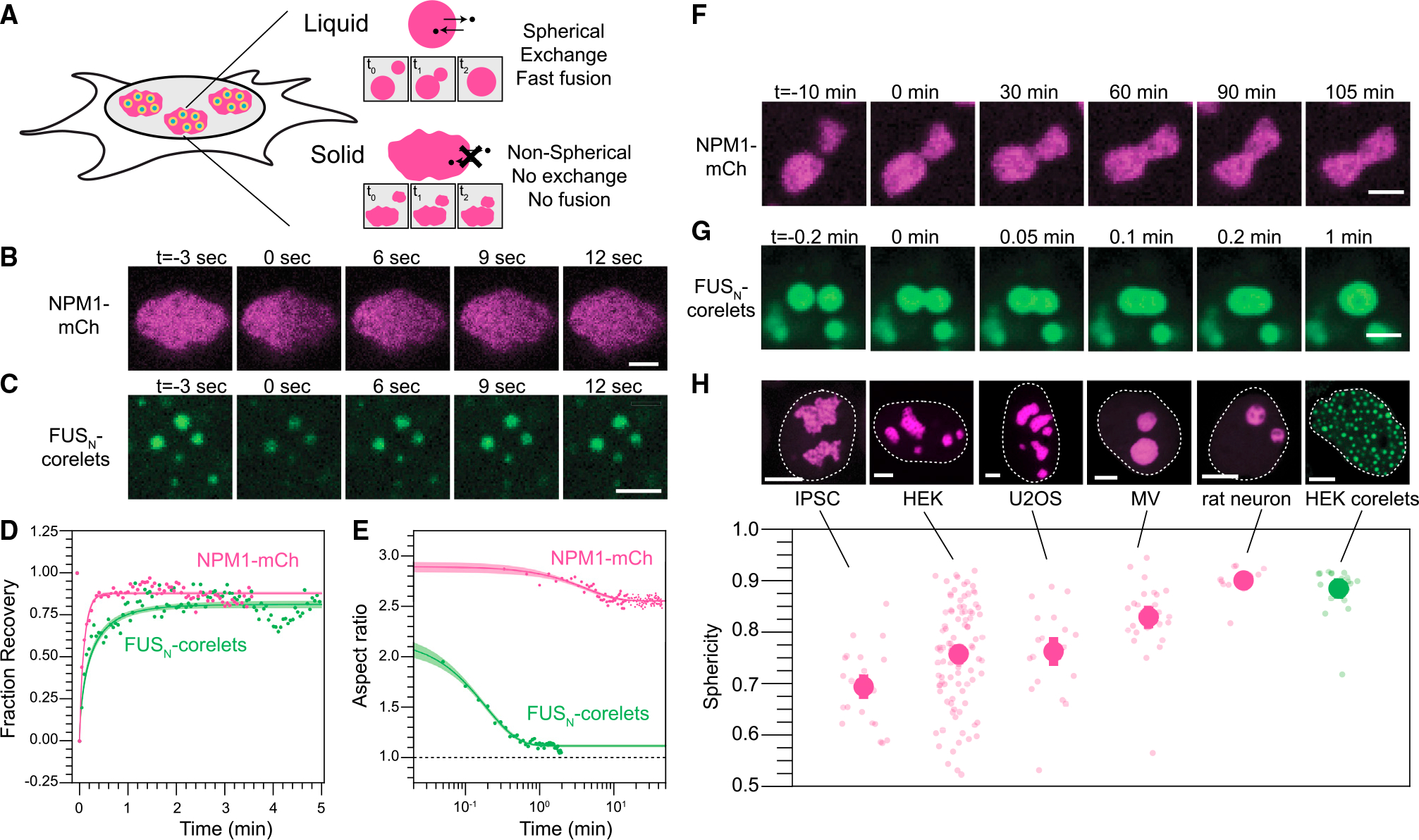
The nucleolus exhibits complex material properties, whereas synthetic nuclear condensates behave like typical liquids. (A) Schematic detailing expected properties of liquid-like vs. solid-like nucleoli. (B) Half FRAP of NPM1-mCh in the GC demonstrates rapid recovery. Scale bar, 2 μm. (C) Similarly, FRAP of multiple small engineered nuclear droplets (FUS_N_-mCh-sspB co-expressing with NLS-GFP-FTH-iLiD^8^) recovers quickly after photo-bleaching. Scale bar, 2 μm. (D) Quantification and averaging of FRAP traces for both NPM1 (half FRAP of individual nucleoli in n = 4 nucleoli in 4 cells) and FUS_N_ (whole FRAP followed by normalization for total fluorophore bleach in 90 condensates over n = 8 cells) reveals recovery on a timescale of seconds. Error intervals are mean prediction bands. (E) The aspect ratio of merging condensates shown in (F) and (G) demonstrates rapid kinetics for engineered droplets but no rounding on a timescale of hours for nucleoli. Error intervals are mean prediction bands. (F) Nucleoli, marked by NPM1-mCh, fuse but fail to fully round even on long timescales. Scale bar, 2 μm. (G) Engineered droplets fuse and round rapidly upon contact. Scale bar, 2 μm. (H) (top) The average morphology of nucleoli ranked from lowest to highest sphericity and engineered droplets. Scale bars, 5 μm. Representative images (top) and quantification of nucleolar morphology (bottom) for multiple cell types. Morphology is quantified by sphericity, where V and A publications are the volume and area, respectively; deviation from 1 indicates nonspherical shape. Error bars show cell-average standard error of the mean with smaller transparent points showing the individual volume-averaged nucleoli for each cell analyzed or each droplet analyzed for the case of HEK corelets. The number of cells (nucleoli) analyzed are 23 (68), 99 (803), 20 (81), 28 (42), and 13 (38) for IPSC, HEK, U2OS, MV, and rat neurons, respectively.

**Figure 2.**
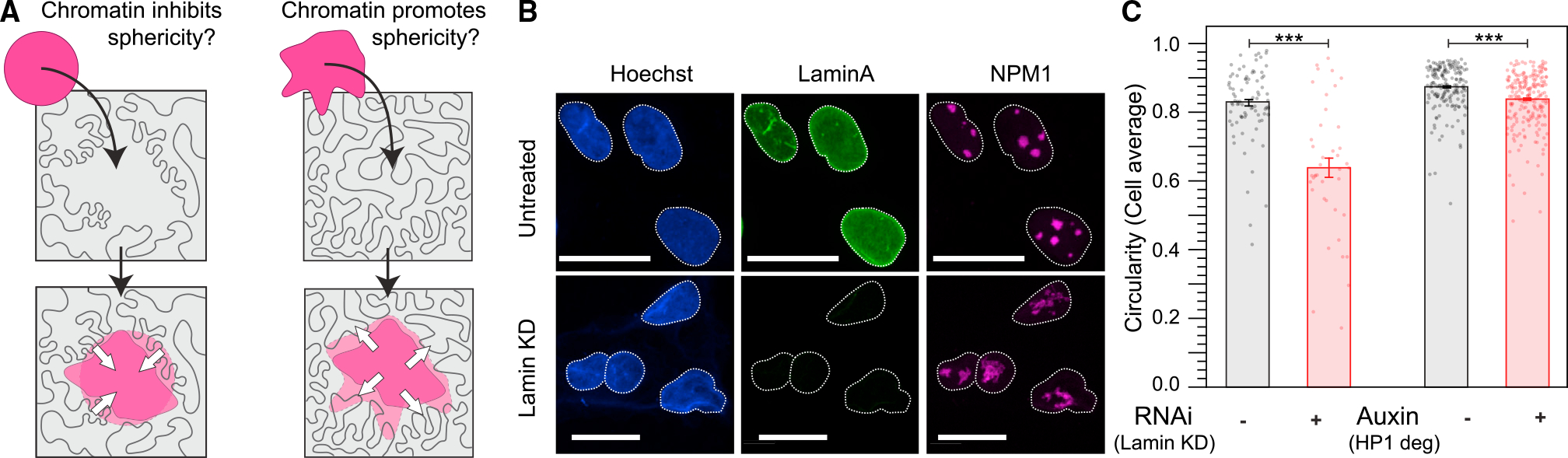
Perturbing the nucleus leads to nucleolar shape changes. (A) Nucleolar shape could be set by constraints from the chromatin container (left) or an intrinsic asphericity (right). (B) Nucleoli get less circular upon siRNA knockdown of Lamin A. Scale bar, 10 μm. (C) Nucleolar circularity decreases upon knockdown of Lamin and degradation of HP1. Significance tested with Mann-Whitney U test. Lamin KD: p = 5 3 10^−8^. HP1 degradation: p = 2 3 10^−7^. Error bars are error on the mean.

To gain insight into how the material properties of the nucleolus facilitate its function, we first re-examined some of the classic dynamic features of nucleoli, which have been used to argue for their simple (i.e., Newtonian) liquid properties.^15,28,30^ Simple liquids and solids are differentiated by their mesoscale dynamics, with liquids showing rapid diffusion, coalescence, and rounding to minimize surface area (Figure 1A). We exogenously expressed mCherry-tagged GC-protein NPM1 by lentiviral infection and followed nucleolar dynamics in HEK293 cells. Consistent with previous studies,^29,30,35,36^ fluorescence recovery after photobleaching (FRAP) experiments show that the nucleolus exhibits fast (<1 min) internal mixing of the scaffold protein NPM1 (Figures 1B and 2D) but slow fusion and frequently aspherical shape in different cell types (Figures 1E, 1F, and 1H); interestingly, continuously dividing cells (e.g., U2OS and iPSCs) tend to have less spherical nucleoli than post-mitotic rat neuron cells (Figure 1H).

These observations indicate that either the local environment or some inherent biophysical feature of nucleoli impedes their relaxation into the spherical shapes generally associated with simple liquid droplets. We reasoned that nonspherical shapes could arise from external mechanical constraints, such as the elasticity of the surrounding chromatin network, similar to water constrained to the shape of a glass. However, we find that optogenetically induced nuclear condensates in HEK293 cells are highly spherical and exhibit fast internal mixing and fusion, consistent with previous studies^8,37^ (Figures 1C, 1D, 1E, and 1G). Nucleoli interact with rDNA regions (NORs) on the genome, whereas FUS_N_ droplets presumably form without interaction with any specific genomic locus. However, in both cases, these condensates are embedded in the crowded nuclear matrix, suggesting that chromatin does not necessarily constrain condensate shape. Indeed, if chromatin were to mechanically constrain nucleolar shape, the effect would have to be specific to nucleoli and not a general constraint on condensates residing within the nucleus.

The chromatin environment around nucleoli is known to be strongly heterochromatic, which could potentially provide an elastically cross-linked, solid-like gel, which might inhibit nucleoli from relaxing into spherical shapes (Figure 2A).^38,39^ To test this, we knocked down heterochromatin protein alpha (HP1α) and Lamin A/C (LMNA), each of which has previously been shown to independently impact heterochromatin organization and nuclear mechanics on a whole-nucleus scale.^40,41^ We reasoned that, if perinucleolar heterochromatin is constraining nucleoli into a nonspherical shape, these perturbations would soften the chromatin and allow nucleoli to round up. However, surprisingly, nucleoli become less spherical in both cases (Figures 2B, 2C, and S1), with statistical significance for both but a larger apparent effect upon LMNA knockdown (Figure 2C). This suggests that the chromatin environment around nucleoli actually helps promote rounding. But it remains unclear why the nucleolus seems to inherently tend toward an aspherical morphology.

### Nucleolar asphericity reflects internal transcription and outward flux of rRNA

**Figure 3.**
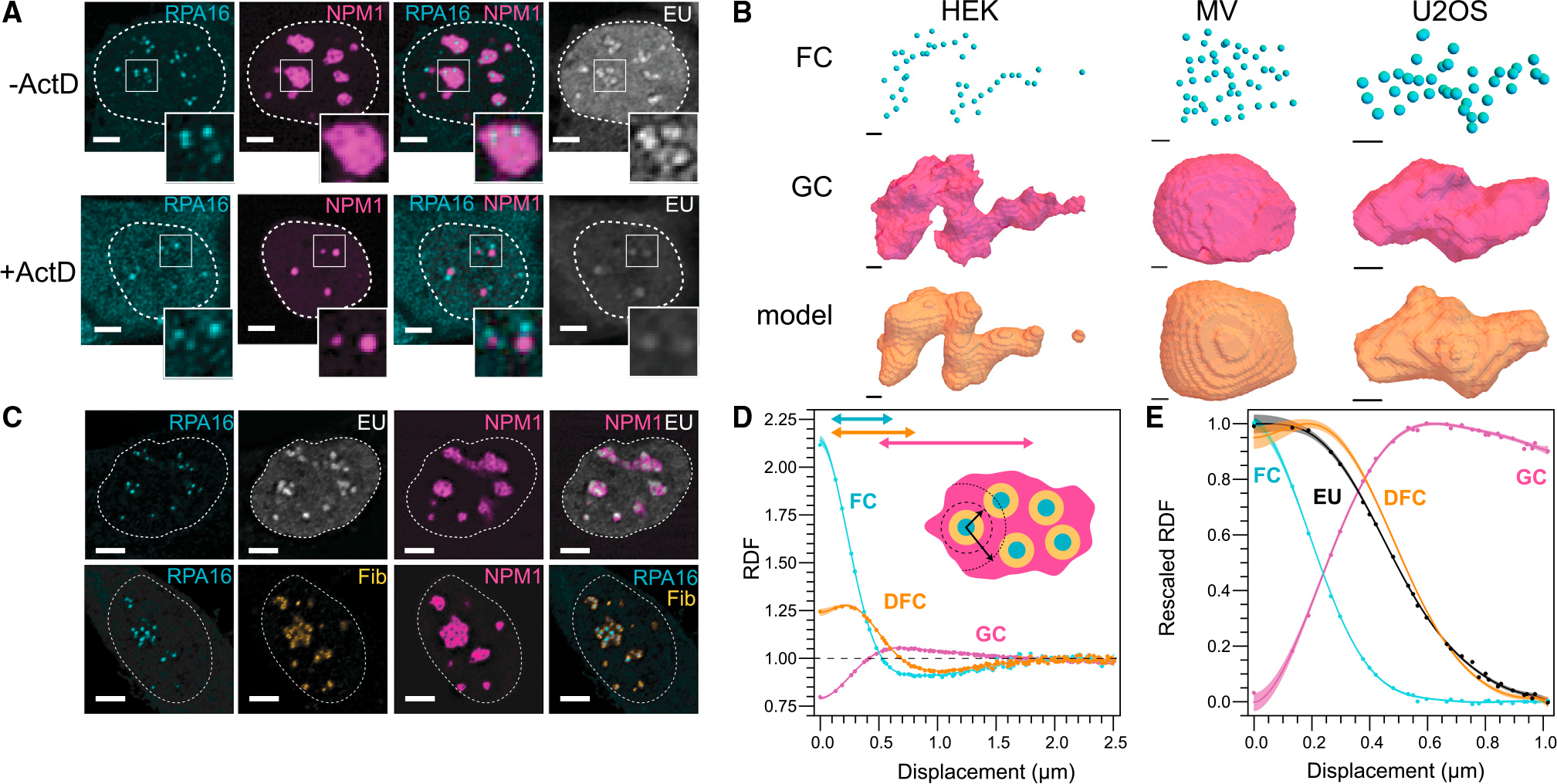
rRNA transcription controls nucleolar shape. (A) Inhibition of RNA transcription by ActD for 4 h results in morphological changes of the nucleolus. Scale bar, 5 μm. (B) Within a simple shape model (STAR Methods), FC location is sufficient to describe nucleolar shape in HEK, MV, and U2OS (left, middle, right). Scale bar, 1 μm. (C) RNA (EU; white) is transcribed at the interface of FC (RPA16; cyan) and DFC (fibrillarin; yellow) (Scale bar, 5 μm. (D) The RDF of nucleolar phases reflects their concentric layering (N = 131 nucleoli). (E) rRNA is primarily located in the DFC 30 min after addition of EU to the media (N = 198 nucleoli); the DFC RDF fit is from (D). Shaded error region on RDFs are mean prediction bands to spline fits (supplemental information).

Having found that the chromatin environment does not cause nucleolar asphericity, but instead actually promotes more spherical shapes, we hypothesized that aspherical morphology may arise from “within,” i.e., as a result of biological activity with the nucleolus. To examine this possibility, we inhibited transcription by adding of the drug Actinomycin D for 4 h. Consistent with previous studies,^42–44^ nucleoli shape changes dramatically. Specifically, we observe nucleolar rounding followed by FCs moving to the periphery, also referred to as “nucleolar caps” (Figures 3A and S2A). To quantify the role of biological activity in governing the shape of the nucleolus, we focused on the movement of rRNA transcription from the surface of the FC outward. This localized production of rRNA produces a radial flux that, at steady state, balances the efflux of material at the surface. We developed this simple transport physics into a model (supplemental information) that predicts the surface of the nucleolus given only the center of the FCs and the total volume of the nucleolus. We compare this with the shape of the nucleolus marked by NPM1 (Figure 3B). This quantification is consistent with rRNA transcripts controlling nucleolar shape because this simple transport-inspired model is sufficient (R^2^ = 0.96 ± 0.01) to describe the overall shape and asphericity of nucleoli prior to inhibition (Figures 3B, S2B, and S2C). Taken together, these data show that transcriptional sources (fixed in space) can give rise to aspherical condensates, supporting the concept that the constant production of rRNA would be anticipated to strongly impact nucleolar shape based on the location of rRNA transcriptional sites.

The strong dependence of nucleolar morphology on rRNA transcription appears at odds with a physical picture of rRNA rapidly diffusing within a purely liquid droplet, whose shape is unconstrained. To gain further insight into the relationship between rRNA and nucleolar structure, we examined nucleolar organization by calculating the radial distribution function (RDF), which is analogous to the pair-correlation function, commonly used to quantify the structure and dynamics of non-living materials.^45,46^ The RDF represents the relative intensity, radially averaged over a specified distance from a particular location, here the center of any FC within the nucleolus; an RDF greater than or less than unity is equivalent to a local spatial enrichment or depletion, respectively. As expected, the concentric nature of the FC (RPA16), DFC (FBL), and GC (NPM1) layers (Figure 3C) is clearly identified through the calculation of the RDF (Figures 3D and 3E). Local minima in the RDFs for the FC and DFC become apparent at ~1 μm, indicating half the average distance between the centers of two FCs (Figures 3D and S3).

Observing the dynamics of rRNA through these layers is challenging due to the lack of *in situ* rRNA labeling strategies. Early biochemical methods took advantage of the abundance of rRNA compared with other RNA transcripts and followed nucleolar RNA via radioactive uridine, or BrU.^42,47^ Building from these early studies, we monitored rRNA through the incorporation of nucleotide 5-ethynyl uridine (EU), which can be visualized following fixation and fluorescent modification of the EU (Figure 3C).^48^ 30 min after EU labeling, images reveal EU signal localized at the DFC phase of the nucleolus (Figure 3C). This is also captured by the calculated RDF for the EU signal, which closely coincides with the DFC curve (Figure 3E), consistent with previous results indicating RNA initially localizes at the FC/DFC interface.^25^ As expected, only a very weak signal is observed after Actinomycin D treatment (Figure 3A). Thus, RDF analysis combined with EU labeling is a quantitative method to determine ensemble-averaged rRNA localization profiles in the nucleolus.

**Figure 4.**
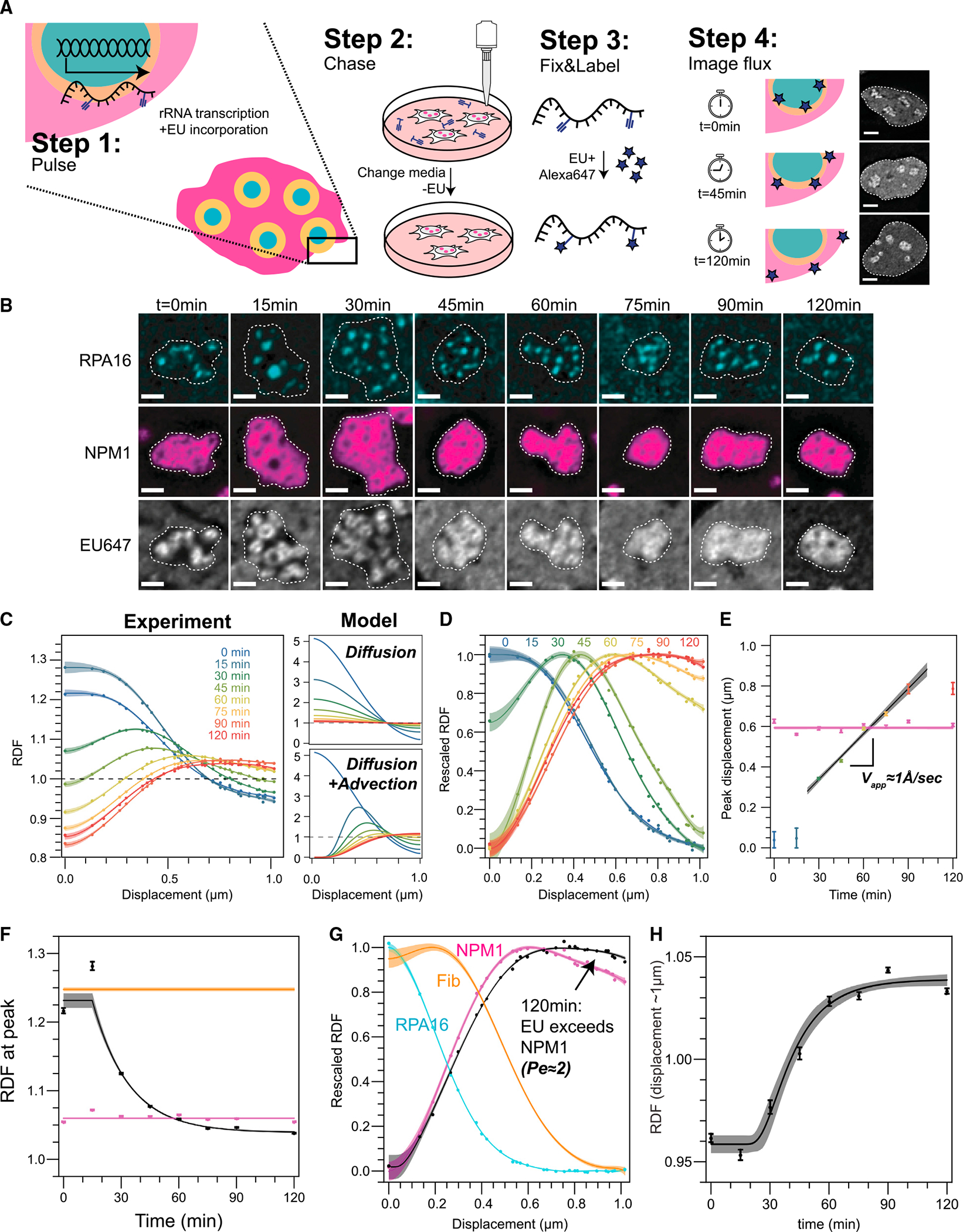
rRNA movement through the nucleolus reflects advective flow. (A) Schematic of pulse-chase EU labeling experiment. Scale bar, 5 μm. (B) RNA (EU; white) moves away from its source (RPA16; cyan) progressively. Dashed lines demarcate individual nucleoli. Scale bar, 2 μm. (C) Left, quantification of RNA peak movement over time (N = 198, 155, 166, 159, 182, 279, 196, and 201 nucleoli for sequential time points). Model, an analytical solution to the spherical advection-diffusion equation, of how the RDF would look in case RNA movement was driven by diffusion (top) or diffusion plus advection (bottom). (D) Rescaled quantification of (C). (E) Maximum enrichment of RNA peak over time (time point coloring as in (C), black line is fit). Maximum NPM1 enrichment remains stable over time (pink points and fit). (F) The RDF at the peak is stable for NPM1 (magenta) and fibrillarin (orange, approximate level shown based on data shown in Figure 3D) but decreases for EU (black). (G) Nucleolar phases and EU rescaled RDF at 120 min, highlighting the EU peak exceeding the NPM1 peak. (H) RDF value for EU signal at ~1 μm from the FC center (corresponding to the edge of the GC), reflecting progressive increase in rRNA concentration at this distance. The EU data in (G) and (H) are fit to the 3D-approximation to the advection-diffusion equation to extract the Peclet number, diffusion constant, and advection; unless otherwise indicated, all other shown curves are spline fits (supplemental information). Error bars and regions are error on the mean and mean prediction bands, respectively.

We next sought to quantify the dynamics of rRNA transport, through a pulse-chase labeling protocol (Figure 4A). Upon incubating cells with EU for 30 min (“pulse”) followed by washout (“chase”) for varying times prior to fixation, we find that the RNA moved progressively away from the FC/DFC (Figures 4B, S4A, and S4B). We quantified these data with the RDF analysis described above. The time-independent RPA16 and NPM1 RDFs indicated both a high degree of reproducibility and a minimal impact of EU on nucleolar form during the experiment (Figures S5A–S5C). In contrast, as expected, the EU RDF changes with time, including a decrease and broadening of the peak of the RDF with time (Figures 4C, 4D, and 4F). Unexpectedly, however, the peak in the signal persisted, and moved radially at a slow apparent velocity of ~1 Å/s (~6 nm/min, or ~0.4 μm/h) with time, a clear indication of motion that is significantly directed, rather than purely diffusive (Figures 4C–4E, S4C, and S4D); interestingly, in cells induced to express higher levels of NPM1, the average GC radius around the FCs increases, accompanied with an increase in rRNA transport dynamics; this is consistent with NPM1’s role as a GC phase separating scaffolding protein and suggests that NPM1 acts as a kind of nucleolar lubricant, accelerating its rRNA outflux (Figure S6). Consistent with considerable directional rRNA motion, solutions to the diffusion equation only exhibit a persistent and shifting peak when advection is included (Figure 4C, right), whereas a simple partitioning model for movement between nucleolar subphases (i.e., rRNA movement from DFC to GC) fails to capture this motion (Figures S5D and S5E). Moreover, we find that the EU RDF at long times is displaced by 0.2 μm (30%) further from the FC than NPM1’s RDF (Figure 4G), which is another signature of substantially directional (i.e., advective) motion that pushes rRNA toward the periphery. These observations suggest that rRNA flow, likely resulting from the large-scale polymerization of nucleotides into rRNA at the FC/DFC interface, drives the slow directional motion of RNA away from the FC/DFC. Nonspherical nucleolar shape thus ultimately appears to result from the effectively fixed locations of FCs, which dictate the pattern of outwardly directed rRNA flux.

When characterizing transport phenomena, the relative strength of advection compared with diffusion is quantified by the Peclet (Pe) number. In the limit of low Pe (Pe << 1), diffusion dominates, whereas in the limit of high Pe (Pe >> 1), advection dominates. To quantify the apparent Pe number, we solve for the 3D-approximation to the advection-diffusion equation as a function of Pe at long times and fit for the ratio of the EU RDF to NPM1’s RDF, which results in a Pe number 2.3 ± 0.1. However, we note that this is a lower limit on the actual Peclet number because the EU signal will be blurred due to experimental constraints, including the diffraction limit of confocal microscopy and delayed kinetics of washout and incorporation of EU even after chase, both of which are expected to increase the apparent diffusivity. Nonetheless, using this estimated Pe number, we can also extend our approximation for the time-dependent solution of the advection-diffusion equation (supplemental information). Analogous to the recovery of a FRAP curve, we focus on the change near the boundary (r ~1 um) to avoid the complications associated with the multiphase structure of nucleoli at lower radial distances and fit to EU RDF to extract an apparent diffusion constant of 0.08 ± 0.01 μm^2^/h (Figure 4H). Employing this fitting analysis on the NPM1 overexpression data shows meaningful increases in both Pe and the diffusion constant, which together result in insignificant changes in advection (Figures S6D–S6M).Taken together, these data all clearly indicate the presence of a significant advective flow of nascent rRNA chains away from their sites of transcription.

### Nucleolar shape and rRNA flux reflect rRNA viscoelastic entanglement

**Figure 5.**
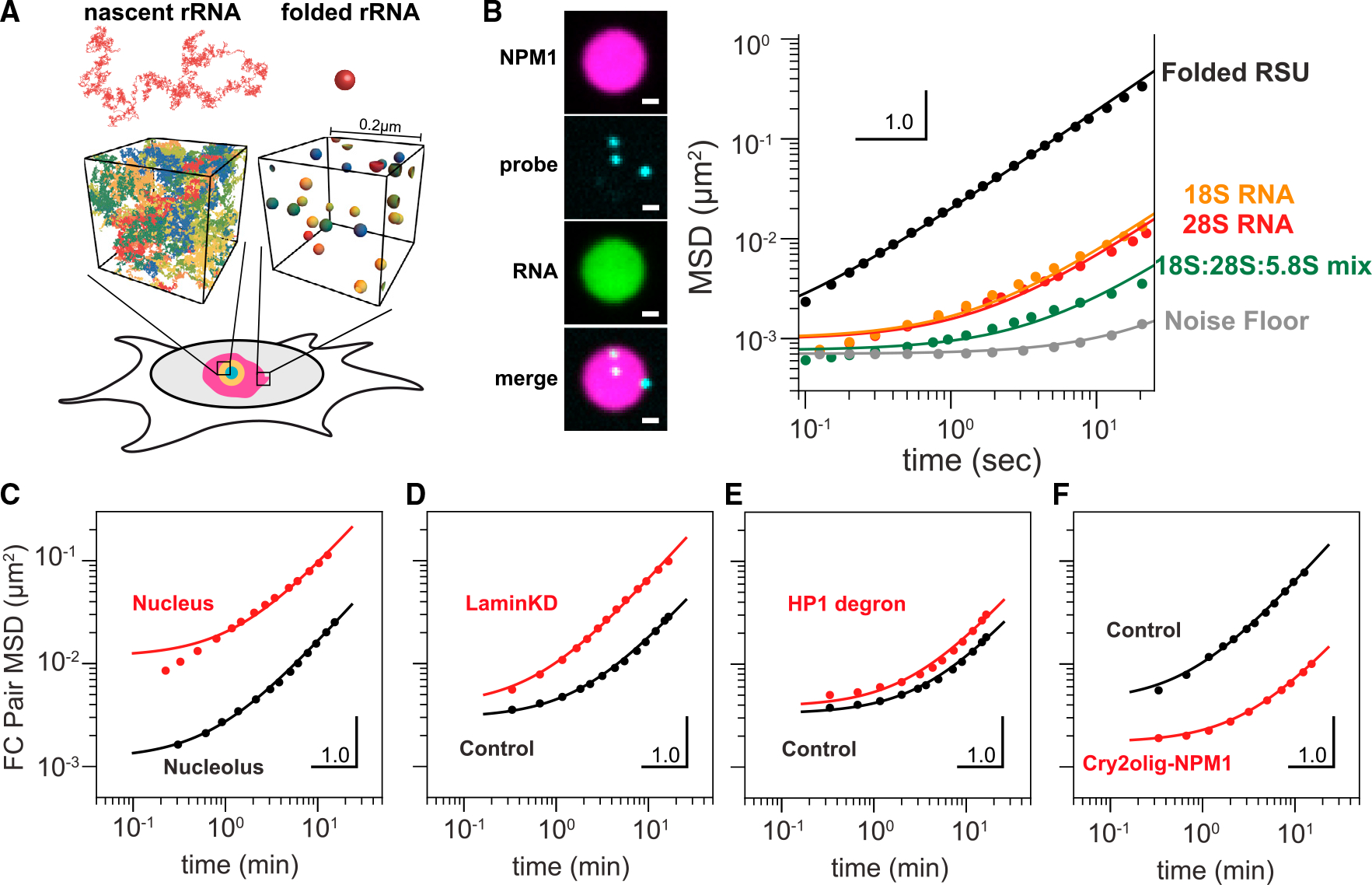
Modeling, concentration estimates and *in vitro* microrheology suggest rRNA-driven changes to nucleolar rheology. (A) Modeled as a random coil, nascent rRNA is ~15-fold larger in diameter than a folded rRNA subunit, suggesting substantial degree of entanglement between nascent chains, but not folded RSUs, using rRNA transcript density estimated from experimental measurements (supplemental information). (B) NPM1 (magenta) and ribosomal RNAs (green) were combined to generate *in vitro* NPM1+rRNA droplets. MSD of microrheological probes in NPM1+rRNA *in vitro* droplets. E. coli rRNAs (black) are purified from mature polysomes. Orange: *in vitro* transcribed 18S rRNA. Red: *in vitro* transcribed 28S rRNA. Green: equimolar mixture of all three *in vitro* transcribed 18S, 28S, and 5.8S RNAs. Gray: noise floor. (C) Pairwise MSD of long-time dynamics of FCs, labeled by RPA16-GFP, in HEK cells (“nucleolus”; 508 pairs of FCs in seven nucleoli) and engineered droplets in U2OS nuclei (“nucleoplasm”; 31,582 pairs in 18 cells, re-analyzed data from.^37^ FCs fit a nonlinear Maxwell model, whereas engineered droplets do not. (D and E) (D) MSD comparing FC dynamics in control vs. Lamin knockdown cells and (E) HP1 degron cells without (black) and with (red) auxin treatment. (F) MSD comparing control cells with Cry2olig-NPM1-activated cells.

We reasoned that such directional flux could arise from entanglement and associated viscoelasticity of nascent rRNA transcripts. Viscoelasticity is common in soft condensed matter, where so-called complex fluids exhibit material properties of both liquids and solids.^49^ Complex fluids include polymeric liquids, where long serpentine polymer chains are interwoven with one another, leading to elasticity on short timescales, and slow viscous relaxation only on long timescales, when chains have time to slide past one another.^49^ Long associative polymers that stick to one another can give rise to additional elasticity or even gelation, resulting in time-independent, solid-like behavior.^50,51^ Remarkably, the nascent rRNA chain is 13-kilobase (kb) long, which corresponds to a total extended contour length of roughly 10 μm, several times larger than the typical diameter of the entire nucleolus.^52^ Consistent with the concept that these long chains should be entangled, calculations suggest that rRNA concentrations may be as high as 10-fold greater than the overlap concentration (Figure 5A; supplemental information), at which separate polymer chains begin to entangle with one another^51^; early electron microscopy studies, indeed, appear to show significant entanglement of nascent rRNA chains.^20^

Given the potential for rRNA to impact nucleolar material (rheological) properties, we sought to determine if the presence of rRNA in reconstituted droplets would impact their rheological properties, using an *in vitro* NPM1 system.^15,29^ NPM1 was mixed with *in vitro* transcribed rRNA or mature ribosomal RNA purified from assembled RSUs, to form reconstituted GC droplets (Figure S7). Probe particles were also added and tracked by confocal microscopy; by calculating their mean square displacement (MSD), probe particles can serve as passive microrheological probes because the mobility of the particles reflects the rheological properties of their environment.^53,54^ Droplets formed with mature RSU rRNA exhibited probe particle MSDs that were linear, with a slope close to 1 on a log-log plot, consistent with the behavior of simple liquids (i.e., no elasticity, Figure 5B, black line). By contrast, droplets formed with *in vitro* transcribed rRNA for the 18S and 28S, or 18S, 28S, and 5.8S added in equal ratios, all showed suppression of probe particle motion compared with the RSU droplets. In particular, the long-time MSD decreased by over an order of magnitude, suggesting a corresponding decrease in the effective viscosity. Interestingly, these data also show significantly nonlinear MSDs at shorter timescales; this nonlinearity can indicate partial elasticity, although we note that the proximity of the noise floor could potentially contribute (Figure 5B). Nonetheless, although the concentration of RNA in these *in vitro* condensates, and thus the degree of entanglement, is expected to be lower than for *in vivo* nucleoli, these data clearly show that the presence of unfolded RNA can dramatically alter material properties and slow down internal dynamics by over an order of magnitude.

Undertaking comparable measurements in nucleoli within living cells is challenging because probe particles are difficult to introduce into the nucleolus. Instead, we relied on the diffraction-limited FC phases as natural probes, whose fluctuating motion can also provide insights into the material properties of nucleoli. To avoid the confounding impact of bulk cellular motion, we quantified the pairwise MSD (pair-MSD) of FCs labeled by RNA polymerase I subunit, RPA16-GFP, i.e., for all pairs of FCs in a particular nucleolus^55^ (Figure S8). We find that the pair-MSD of nucleolar FCs is small and strongly nonlinear, exhibiting subdiffusion on short timescales (Figure 5C, black line); we note that these data, particularly at short timescales, can also be impacted by the proximity of the noise floor. However, this motion contrasts with that of synthetic condensates elsewhere in the nucleus, which move significantly faster (Figure 5C, red line). Moreover, upon loss of HP1α or LMNA, FC motion is increased (Figures 5D and 5E). This is consistent with increased chromatin dynamics, whereas the overall curvature (nonlinearity) is unchanged (Figures 5D and 5E). In contrast, increasing cross-linking/entanglement in the nucleolus with our previously published optogenetic approach^56^ decreases FC motion and substantially increases the overall MSD curvature (Figure 5F). In all these cases, FC motion (or probe motion in Figure 5B) in the nucleolus can be well-fit to a Maxwell fluid model describing motion inside viscoelastic polymer melts,^57,58^ whereas the subdiffusive motion of chromatin loci outside of the nucleolus cannot (Figure 5C, red curve). Together, these data are consistent with significant viscoelasticity in the nucleolus due to entangled nascent rRNA transcripts.

**Figure 6.**
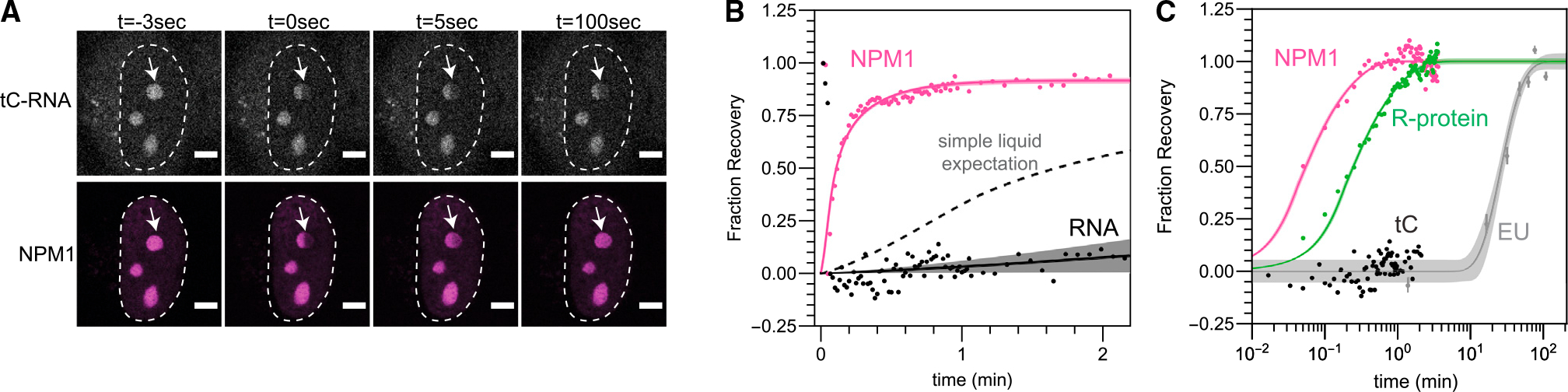
RNA and nucleolar proteins exhibit vastly different dynamics. (A) RNA labeled with tC nucleotides does not recover after bleaching of half of the GC, whereas NPM1-mCh exhibits full recovery. (B) FRAP recovery curve for tC (black) and NPM1 (pink). Dotted line represents expectation for RNA recovery if the nucleolus were a simple liquid. (C) NPM1 (pink), R-proteins (green), and RNA (EU in gray line, tC in black dots) have different dynamics. Shadded error regions are mean prediction bands.

If entanglement and viscoelasticity underlie the slow directional motion of nucleolar rRNA (Figure 4), it may be unclear how nucleolar proteins such as NPM1 could simultaneously exhibit rapid dynamics (Figures 1B and 1D), which is often mistakenly interpreted as reflecting a purely liquid material state. To underscore this point, we note that the measured diffusion constants of NPM1 (Figures 1B and 1D) and rRNA (Figure 4H) are 0.1 μm^2^/s and 2*10^−5^ μm^2^/s, respectively; this ~5,000-fold difference is much larger than the ~15-fold difference anticipated for diffusion in a simple liquid. To further probe and validate these major differences in mobility, we sought to directly perform comparable half-FRAP mobility measurements for both rRNA and nucleolar proteins. To achieve live-cell imaging of rRNA, we deployed a cutting-edge method to incorporate the fluorescent cytidine analog 1,3-diaza-2-oxophenothiazine (tC) into RNA using human cells expressing uridine-cytidine kinase 2 (UCK2)^59^ (Figure 6A). In cells labeled with both tC and NPM1-mCherry, as expected, locally photobleached NPM1 exhibits rapid FRAP recovery. Local photobleaching of RNA, however, does not exhibit significant FRAP recovery, even after several minutes (Figure 6B, black line), in stark contrast to the recovery expected for a simple liquid of the size of rRNA (Figure 6B, dotted line). Utilizing our EU data (Figure 4) to calculate an equivalent FRAP recovery curve for rRNA (STAR Methods), we find that the characteristic timescale for molecular “FRAP recovery” is ~3 orders of magnitude slower for rRNA compared with NPM1; this is consistent with the vast differences in dynamics recently observed for nascent rRNA and NPM1 mixtures *in vitro*.^60^ The live-cell tC-labeled data are consistent with our EU data, with the former closely following the early time points of the EU experiment (Figure 6C). Thus, although it appears that rRNA entanglement makes the nucleolus a slowly relaxing viscoelastic fluid, nucleolar proteins readily diffuse, likely due to their ability to diffuse through the interstices of the RNA mesh.^56^

**Figure 7.**
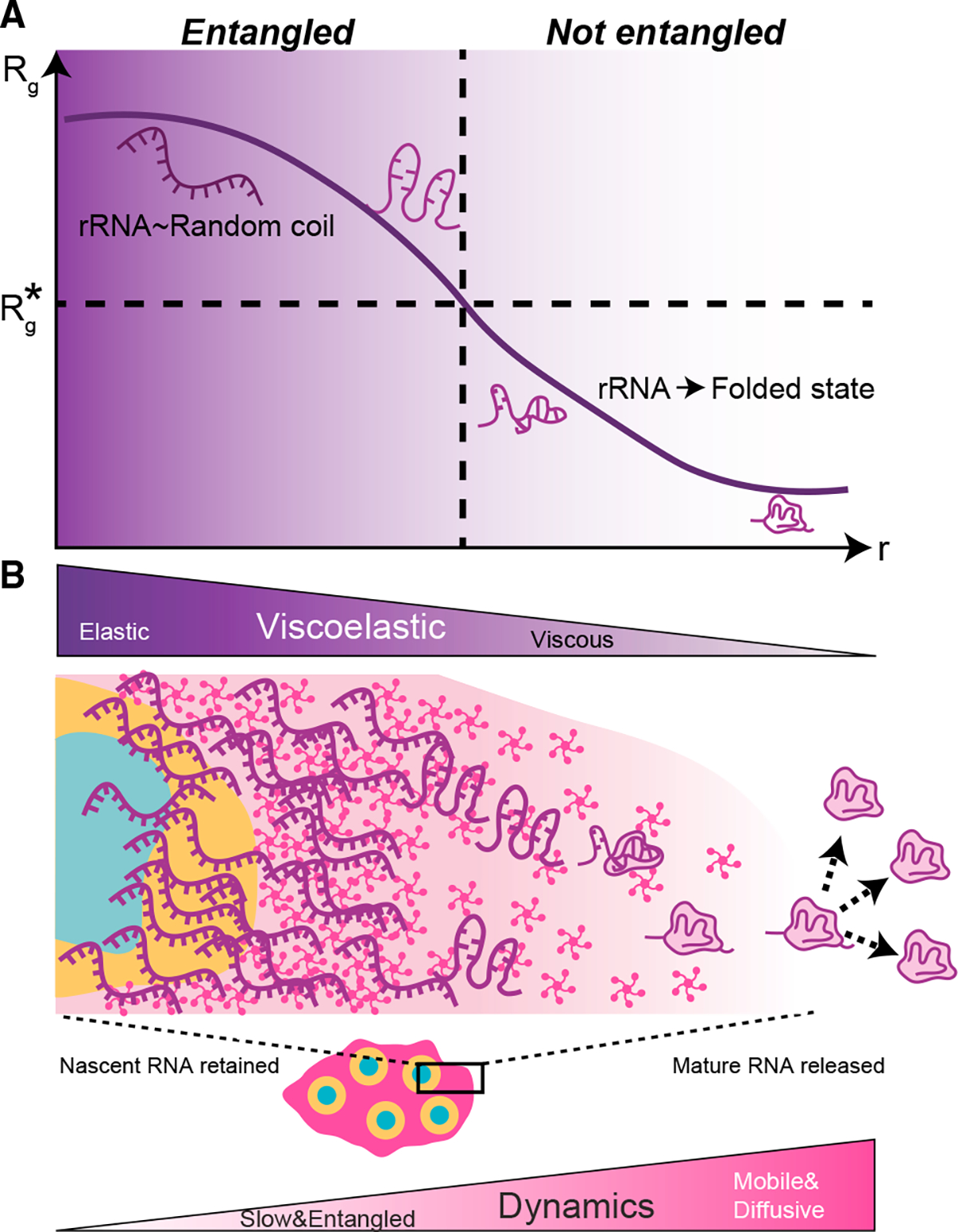
Model for a gradient of viscoelasticity underlying the assembly line nature of ribosome biogenesis in nucleoli. (A) Schematic depicting changes in rRNA conformation, which leads to a progressive decrease in the effective size of rRNA (i.e., radius of gyration, Rg), dropping below the size (Rg*) at which chains are no longer overlapping and entangled. (B) Schematic of nucleolar viscoelasticity during the progression of ribosome subunit assembly; mature pre-ribosomal particles move more diffusively through the peripheral GC liquid and are released into the nucleoplasm. Conformations of rRNA and/or ribosomal subunits are shown as purple graphics, whereas pink pentamers represent nucleolar phase separating proteins such as NPM1.

Importantly, our FRAP data for rRNA represent an average over its different processing/conformational states; we interpret these data as primarily reflecting the slow, entangled nascent chains, although the mobility of mature, folded chains is likely significantly faster. Indeed, once folded chains leave the nucleolus, the only way to replace those newly departed chains is through the slow flux of maturing chains moving radially outward. This physical picture implies that, as rRNA progressively matures and folds into compact ribosomal subunits, not only does its valence decrease,^15,61,62^ but it should also become less entangled. Consistent with this physical picture, a simple calculation suggests that fully processed and assembled rRNA chains will overlap with themselves over 3,000-fold less compared with the nascent chains (Figure 5A; supplemental information). To test this concept, we performed FRAP experiments on the ribosomal protein RPL5. Despite monomeric RPL5 being substantially smaller than pentameric NPM1, RPL5 exhibits considerably lower mobility (~5-fold) compared with NPM1 (Figure 6C), suggesting much of the nucleolar RPL5 is incorporated with rRNA into the RSU.^63^ However, the mobility of RPL5 is nonetheless several orders of magnitude larger compared with the average RNA signal, as would be expected if RPL5 mobility reflected that of late-stage mature rRNA (Figure 6C). In further agreement, a near doubling of the nucleolar volume due to NPM1 overexpression (Figure S6I) has a modest doubling of the diffusion constant (Figure S6L) that is consistent with a decrease in entanglement anticipated from the dilution of rRNA. This is consistent with a physical picture in which ribosomal proteins bind to and help the progressive folding of rRNAs, which is associated with a decrease in their size and degree of entanglement with other rRNAs (Figure 7).

## DISCUSSION

In many systems, the nucleolus and other biomolecular condensates are observed to have some liquid-like features, including rapid protein dynamics, concentration-dependent organelle size, and relatively spherical shape.^28,30,53^ However, materials scientists have long appreciated that fluids containing long polymers, often referred to as complex fluids, can exhibit rheological properties of both solids and liquids, known as viscoelasticity.^46,49,64,65^ Our findings suggest that the nucleolus is a complex fluid with viscoelastic properties, supported by the following: (1) the intrinsic asphericity of nucleoli, even after relieving constraints from the surrounding heterochromatin, (2) the slow radial movement of rRNA away from its source, which is inconsistent with pure diffusion through a liquid, (3) microrheological measurements of *in vitro* reconstituted nucleoli and FC dynamics in live cells, exhibiting features characteristic of high viscosity and/or partially viscoelastic materials, and (4) EU pulse-chase and live-cell nucleotide imaging experiments revealing that the dynamics of nascent rRNA chains are extremely slow, with effective FRAP recovery times measured in tens of minutes, rather than tens of seconds for typical nucleolar proteins. Taken together, our data clearly show that the nucleolus is a complex fluid with rheological properties underlying its form and function.

Given previous findings from our group and others, on the liquid-like behavior of nucleoli and *in vitro* nucleolar facsimiles, nucleolar viscoelasticity may be unexpected. However, deviations from purely liquid-like nucleolar behavior have been suggested in previous work, including aging and viscoelasticity of condensates formed from fibrillarin,^30^ activity-dependent coalescence dynamics of nucleoli in xenopus oocytes,^28^ and the surprisingly slow relaxation dynamics of coalescing mammalian nucleoli.^38^ Moreover, several recent studies underscore how RNA-RNA and RNA-protein interactions can result in solid-like material properties.^4,60,64,66,67^ Thus, our findings add to a growing body of evidence showing how RNA can give rise to complex rheological behaviors.

Previously proposed models have emphasized the thermodynamic driving forces for nucleolar form and function. By contrast, our data show the critical importance of transport kinetics, which we interpret as being dictated by RNA entanglement and viscoelasticity. This physical picture of partially extended and interwoven ribonucleoprotein chains may at first seem in conflict with cryoEM structures of individual ribosomal subunit intermediates.^26^ However, such collapsed intermediates are primarily observed at a gradient near the surface of the nucleolus and lack ~50% rRNA density, much of which is contained in ~250-bp stretches.^27^ Indeed, early electron microscopic images of nascent rRNA chains in amphibian oocytes show a high density of long, partially overlapping chains.^20^

We note that physical origins of the very slow observed RNA dynamics other than chain entanglement are possible, such as gelation imparted by high multivalency.^56,66^ Such partially arrested, “glassy dynamics” are seen in soft matter systems whose constituents are not entangled polymers.^68^ But given the presence of high concentrations of long nascent RNA chains in the nucleolus, with our calculations suggesting they are above the overlap concentration and clearly moving in a very slow and directional fashion, our findings collectively point to RNA entanglement as a key feature.

One fascinating aspect of our findings is that nucleolar proteins show substantially faster dynamics compared with the much slower mobility of rRNA. This suggests that, although rRNA forms a viscoelastic gel, it is enmeshed within and surrounded by a more dynamic and partially liquid-like protein fluid. We speculate that such an environment could be conducive to snoRNA- and protein chaperone-mediated regulation of rRNA folding, as observed in previous studies.^69,70^ Further, this kinetic and material-based model could explain how that process is spatiotemporally regulated during the modular steps of ribosome biogenesis.^26^ Indeed, our physical picture suggests that the nucleolus will exhibit spatially dependent viscoelasticity, with the most peripheral regions of the GC, farthest from nascent transcriptional sites at the FC/DFC interface, likely exhibiting behavior closest to that of a purely liquid condensate. Precisely mapping these spatial variations within nucleoli and other condensates will be challenging due to the limited technologies available for unambiguous intracellular microrheological mapping, but it represents an exciting future frontier.

An essential challenge of the phase-separation field is to elucidate the connection between condensate form and function, i.e., linking the biophysical processes underlying the formation and properties of condensates with functional biochemical processes that occur within them. Prior to this work, the form and function of the nucleolus were only suggestively connected, with the concept of the nucleolus as a purely liquid condensate providing limited insight into the mechanisms behind the highly coordinated, sequential, and efficient process of ribosomal subunit assembly. Our findings suggest a simple but powerful physical picture in which these sequential processing steps are mediated by kinetic trapping of incompletely processed rRNA within a partially gelled meshwork. This implies that sequential processing steps progressively relieve kinetic barriers to rRNA mobility and ultimately allow for binding of rRNA to ribosomal proteins and their release into the nucleoplasm as mature ribosomal subunits (Figure 7). Our study provides much needed links to bridge the biophysics of phase separation and related phase transitions with the functional biochemistry of cellular compartmentalization.

This concept of “viscoelastic release” provides a physical model for the controlled and gated motion of rRNA out of the nucleolus. Indeed, a critical aspect of ribosome biogenesis may be the suppression of RNA mobility relative to various accessory factors as well as the ribosomal proteins that rRNA binds to and together form stable members of the ribosome. It is tempting to speculate that this mechanism also serves to prevent local rRNA kinetic trapping into non-native structures that can compete with proper ribonucleoprotein complex and RNA tertiary structure formation^71–73^ and allows for chaperone-assisted preclusion of premature rRNA folding that can be detrimental to ribosome assembly.^70^ The ~5,000-fold faster diffusivity of NPM1 compared with that of rRNA implies that proteins are able to fully sample their phase layer (i.e., GC) of the nucleolus, all while nascent rRNA is effectively frozen, awaiting sufficient processing and disentanglement. With sufficient assembly and movement toward the peripheral GC layer, rRNA eventually becomes more mobile and diffusive. At this stage, the quasiequilibrium physical picture of the liquid-like condensate environment becomes more appropriate, with associated thermodynamic exclusion of folded ribosomal subunits.^15^

In conclusion, our findings provide a striking example of how condensate material properties and rheology are intimately linked to function. The physical picture of the nucleolus that is emerging underscores that it is a particularly sophisticated and complex condensate, whose formation reflects a non-equilibrium steady state, linked in non-trivial ways to the surrounding chromatin environment (e.g., Figure 2), and with spatiotemporally varying biological processes functionally coupled to associated viscoelastic material properties. This complexity represents an exciting opportunity for completely new types of physics-based modeling that build on, but ultimately extend far beyond, the simplest conceptual frameworks of biomolecular phase separation and gelation.^4,74–76^

### Limitations of the study

Our pulse-chase EU results do not fully explain how each individual rRNA molecule moves throughout the nucleolus. This arises due to spatial resolution limits of light microscopy (confocal), the fact that the EU signal does not discriminate between separate RNA chains, and an analysis that isotropically averages spatial movement. How the complex pattern of active rDNA loci and location of multiphase nucleolar compartments (FC and DFC) contribute to the precise movement of an rRNA molecule still needs to be further explained. Moreover, although our results indicate that chromatin dynamics do not contribute significantly to nucleolar asphericity, we cannot exclude that this will be true in all biological contexts or that specific chromatin regions may increase asphericity (e.g., nucleolar-associated domains). Additionally, the use of the FC as a probe to measure (passive) rheology of the nucleolus must be considered with associated caveats, including the active nature of rRNA transcription at the FC and the location of the noise floor. Finally, although the remarkably slow diffusion of rRNA in the nucleolus is clear and a central feature of ribosome biogenesis, the precise contributions of physical entanglement of individual rRNA chains, trans-RNA-RNA hybridization, protein-mediated chain cross-linking, and associated RNA network gelation require further investigation.

## STAR★METHODS

### RESOURCE AVAILABILITY

#### Lead contact

Further information and requests for resources and reagents should be directed to the lead contact, Clifford Brangwynne (cbrangwy@princeton.edu).

#### Materials availability

All reagents generated in this study will be made available on request.

#### Data and code availability

- Microscopy data in this paper will be shared upon request.
- Original code will be shared upon request.
- Any additional information required to reanalyze the data reported in this paper is available upon request.

### EXPERIMENTAL MODEL DETAILS

#### Cell culture

HEK293 (referred as HEK in the manucript), U2OS, and MV (Monkey Vero) cells were cultured in 10% FBS (Atlanta biological S1150H) DMEM (GIBCO 11–965-118) supplemented with penicillin and streptomycin (Thermo Fisher 15140122) at 37°C with 5% CO_2_ in a humidified incubator. For passaging and imaging, cells were dissociated from the plate with trypsin (Trypsin-EDTA 0.05%, Fisher Scientific 25300054) and transferred to 96-well glass bottom dishes (Thomas Scientific) coated with fibronectin.

#### Rodent neuron cell culture

For rodent cortical neuron culture, cortex was dissected from E17 Sprague-Dawley rat embryos (Hilltop Lab Animals Inc.), and neurons were dissociated into single cells using the Worthington Biochemical papain dissociation system. Briefly, cortices were incubated in 5 mL papain solution (20 units/mL papain, 1 mM L-cysteine, 0.5 mM EDTA, and 200 units/mL DNase in HBSS) in a 37C water bath for 20 min with no agitation. Supernatant was discarded and replaced with 3 mL inhibitor solution (1 mg/mL ovomucoid protease inhibitor, 1 mg/mL bovine serum albumin, and 167 units/mL DNase in HBSS) for 5 min at room temperature. Supernatant was discarded and replaced with another 3 mL of inhibitor solution for 5 min at room temperature. Supernatant was removed and 1.5 mL of Gibco Neurobasal Plus complete media (2% B27 Plus, 1% penstrep, 250 ng/mL Amphotericin B) was added. A flame treated pasteur pipette was used to dissociate the tissue by pipetting up and down 10 times, cell clumps were allowed to sink for 1 min, and 750 μL of dissociated cells were removed from the top and added to a new tube for subsequent steps. 750 μL more neurobasal media was added to the remaining clumped cells in the old tube, and the trituration procedure was repeated for a total of 3 dissociation steps, with all of the media being moved to the new tube after the final step. Cells were centrifuged 5 min 300g. Supernatant was discarded, and cells were resuspended in 1 mL neurobasal media for cell counting. Cells were plated with 1x CultureOne supplement (Gibco) in neurobasal media to kill glial cells. CultureOne supplement was only used in media on DIV0 (day in vitro 0), and not used in subsequent media changes. 80,000 neurons were plated per well (~40,000 cells/cm2) in 24 well glass bottom plates treated with Poly D Lysine (0.01 mg/mL overnight treated at 37C, washed x4 in PBS with no drying steps). Half of the media in each well was exchanged for fresh neurobasal media every 3–5 days. Lentivirus infection was done ~DIV13, and cells were imaged once fully mature, DIV17–21.

#### iPSC cell culture

Induced pluripotent stem cells were obtained from Allen Institute for Cell Science at the Coriell Institute. The iPSC line AICS-0084–018:WTC Dual tagged FBL-mEGFP/NPM1-mTagRFPT-cl18 (mono-allelic tags) was used for our experiments. The colonies were expanded and maintained on Matrigel (Corning) in mTeSR Plus medium (Stem Cell Technologies). Cells were plated at 3000–10.000 cells per square centimeter in order to obtain ~75% confluency every 5–7 days. The cells were passaged using ReLeSR (stem cell technologies) and split at a 1:10–1:50 ratio. mTeSR plus medium was supplemented with ROCK inhibitor Y-27632 (Selleckchem) for maximum 24 hours after cryopreservation or passaging. iPSCs were cryopreserved in mTeSR Plus medium supplemented with 30% Knock Out Serum Replacement (Gibco Life Technologies) and 10% DMSO.

### METHOD DETAILS

#### Plasmid construction

DNA encoding NPM1 (Sino Biological) and RPA16 were amplified with PCR using primers synthesized by IDT. Resulting fragments were cloned into linearized FM5-mCh or FM5-GFP constructs using In-Fusion Cloning kit (Takara). The resulting plasmids were sequenced to confirm correct insertion.

#### Lentiviral transduction

Using our mCh and GFP constructs, we created stably expressing cell lines transduced with lentivirus. Lentivirus was produced by transfecting the desired construct with helper plasmids PSP and VSVG into Lenti-X cells with Fugene HD transfection reagent. Virus was used to infect cell lines in 96 well plates. Three days after addition of virus, cells were passaged for stable maintenance. For rodent neurons, third generation lentivirus production was performed with standard protocols. Virus was concentrated using Lenti-X Concentrator (Takara) and resuspended in DPBS before being applied to neurons.

#### Immunofluorescence DFC

Cells were fixed by adding 4% formaldehyde to the wells. After 10 minutes, cells were washed with wash buffer (0.35% Triton-X, Thermo Fisher PRH5142, in PBS, Thermo Fisher 14190250), and permeabilized with 0.5% Triton-X in PBS for 1 hour. Cells were then blocked for 1 hour with 10% goat serum in TBS-T (20mM Tris, 150mM NaCl, 0.1% Triton-X). The primary antibody (Rabbit polyclonal anti-fibrillarin, Abcam 5821) was dissolved in blocking buffer at 0.1μg/ml and incubated overnight at 4°C. The next day, the cells were washed three times with TBS-T. The secondary antibody (AlexaFluor 647 goat-anti rabbit Thermo Fisher A-21245, 1:1000) was dissolved in blocking buffer and incubated for 2 hours at room temperature. Finally, cells were washed three times with TBS-T.

#### EU labeling

For labeling transcribed RNA, the Click-iT RNA imaging kit was used (Thermo Fisher C10330). Largely, the manufacturer protocol was used, with the following adaptations. We performed the protocol in 96-well plates, with volumes adjusted accordingly. Throughout, we kept the volumes at 100μl per well. We prepared the EU solution at 2mM, and 100μl to the 100μl media already in the well, for a final concentration of 1mM. We kept the incubation of EU with the cells constant at 30 minutes. Next, we removed the media containing EU with fresh media not containing EU (for the pulse experiments). For fixation, we added 66μl of 16% formaldehyde in PBS to each well, and incubated for 15 minutes. This was followed by permeabilization with 0.5% Triton-X for 15 minutes. Addition of the Click-iT reaction cocktail per manufacturer instructions, with the exception that we use Alexa-647 (Alexa Fluor 647 Azide, Triethylammonium Salt, ThermoFisher A10277) instead of the fluorophore supplied with the kit. Reaction cocktail was incubated for 30 minutes, cells were washed once with the kit-supplied rinse buffer, and once with PBS before proceeding to imaging. For the temperature variation experiments, cells were incubated for 30 minutes with EU as described above to ensure proper incorporation. Only after EU incubation were cells moved to incubators at different temperatures.

#### Actinomycin D and CX treatment

HEK cells were treated for 4 hours with media containing 1 μg/mL actinomycin D (Sigma, A5156–1VL) dissolved at 0.5 mg/mL in DMSO (Sigma). Control cells were treated with DMEM containing DMSO. EU labelling was performed as described above in media containing actinomycin D or DMSO.

For CX, HEK cells with 1μM/10μM final concentration of CX-5461 (Selleckchem, S2684, dissolved in 50 mmol/L NaH_2_PO_4_(pH 4.5)) RNA Polymerase I inhibitor in DMEM for 4 hours. Control cells were treated with the equivalent amount of solvent (NaH_2_PO_4_(pH 4.5)) used in the drug treatment.

#### Corelet activation and FRAP

Cells expressing the two Corelet constructs^8^ were imaged on a Nikon A1 laser scanning confocal microscope with a 100x oil immersion Apo TIRF objective (NA 1.49) and a Nikon Eclipse Ti2 body; activation was performed by imaging with the 488 laser. Following 5 minutes of activation, droplets were bleached using the 561 laser in a small region of interest and imaged at 3 seconds per frame for 5 minutes. For nucleolar FRAP, cells coexpressing NPM1-mCherry and RPL5-GFP were bleached using the 561nm laser for NPM1 and the 488nm laser for the RPL5 and imaged in both channels. For nucleolar half-FRAP experiments with tC-RNA and NPM1, HeLa cells overexpressing Uck2 kinase and NPM1-mCherry were imaged in FluoroBrite^™^ media after a 12 hour chase with tC nucleotide as previously described.^59^ Experiments were performed on a Nikon A1R resonant scanning confocal microscope with a 60x oil immersion Plan Apo Lambda (NA 1.4); nucleoli with tC nucleotide (RNA) and NPM1-mCherry were bleached using the 405 and 561 nm lasers, respectively, and imaged in both channels.

Images were analyzed in Fiji (ImageJ 1.52p)^81^ and MATLAB 2019b (Mathworks). For corelets, droplets were segmented in the 488 channel; their intensities were averaged and normalized with 1 set to the frame before bleach and 0 set to the frame immediately after bleach; recovery was further normalized to non-bleached control ROIs of the same cells. For nucleoli, each nucleolus was segmented (*imbinarize*) using the non-bleached channel and the intensity profile was calculated for each nucleolus along the axis perpendicular to the half-FRAP, normalizing by the average intensity over the nucleolus. Recovery was then measured as the intensity in the half of the nucleolus that was bleached and normalized with 1 set to the frame before bleach and 0 set to the frame immediately after bleach.

#### NPM1-Cry2Olig Activation

We used an NIH-3T3 cell line in which NPM1 is endogenously labeled with mCh-Cry2Olig at the C-terminus using CRISPR-Cas9.^56^ Cells were exposed to blue light for 15 minutes to photoactivate Cry2olig. To indicate gelation, we performed FRAP on the activated nucleoli (bleached 1μm^2^ spot and tracked recovery for 90 seconds) and checked that recovery was <10% to confirm gelation. We then tracked displacement of FCs marked by RPA16-GFP as described under “MSD tracking”.

#### EU and immunofluorescence imaging

EU and immunofluorescence stained cells were imaged on a Nikon A1 laser scanning confocal microscope using an oil immersion objective, Plan Apo 60X/1.4NA. Imaging conditions were optimized to increase signal to noise. Proteins tagged with GFP were imaged using a 488nm laser, mCherry with 560nm, and Alexa 647 with 640nm. Images shown in Figures 3A, 3C, 4B, S4A, and S4B were deconvolved using the Richardson-Lucy algorithm in the Nikon Elements software V4.40. All example images are optimized to show the full pixel intensity range, with the exception of Figure 3A in the EU channel.

#### HP1 and Lamin degradation experiments

HP1-AID cells are U2OS with both endogenous CBX5 (HP1alpha) allele tagged on C-term with eGFP and the auxin-inducible degron (cell line developed in^40^). Cells were treated with fresh media with no additions (control) or with 1 mM auxin (Indole-acetic acid IAA sodium salt, sigma I5148–2G) 16–24 hours prior to fixation.^82^ Cells were fixed with 4% PFA for 15 minutes at room temperature, washed with PBS 3 times for 5 minutes each, permeabilized in 0.2% PBST for 30–60 minutes, blocked with 5% normal goat serum in 0.1% PBST, and stained overnight with Alexa Fluor 647-conjugated mouse anti-NPM1 antibody (1:1000, ThermoFisher Scientific, Cat. #MA3–25200-A647). One hour prior to imaging, sample was washed 3 times for 5 minutes each with PBS then incubated with 1:5000 Hoechst DNA stain (ThermoFisher Scientific, Cat #62249) for 30 minutes.

Lamin A knockdown was performed on U2OS cells at 30–50% confluency by Lipofectamine RNAiMAX (Invitrogen) delivery of an anti-Lamin A RNAi construct (Ambion, #4427038). Control cells were treated with RNAiMAX reagents containing no RNAi, or an off-target Cy5 labeled control siRNA (SignalSilence^®^ Control siRNA Cy5^®^ Conjugate #86921).

Fixation, permeabilization and blocking was performed as described above. Overnight staining was done with Alexa Fluor 647-conjugated mouse anti-NPM1 antibody (1:1000) and anti-LaminA/C (1:1000, Active Motif 36287). The next day, sample was washed 3 times for 5 minutes each with PBS, then incubated with Goat-anti-mouse-Alexa Fluor 488 (1:1000, Thermo Fisher A-11029) and Hoechst (1:5000) for 2 hours. Sample was washed 3 times for 5 minutes each.

Immunofluorescence samples were imaged on a spinning-disk confocal microscope (Yokogawa CSU-X1) with a x100 oil immersion Apo TIRF objective with 1.49 NA and an Andor DU0897 electron-multiplying charge-coupled device (emCCD) camera on a Nikon Eclipse Ti body. Z-planes throughout the nucleus were collected at 0.5 micron step size and sample imaged with 405 (Hoechst), 488 (eGFP), and 647 (Alexa 647) lasers.

#### *In vitro* transcription of rRNA species

Ribosomal DNA (rDNA) templates encoding 18S, 28S, and 5.8S ribosomal RNA were amplified from cDNA purified from HEK 293T cells with forward primers containing the T7 RNA polymerase promoter sequence (GGATTCTAATACGACTCACTATAGGG). 5.8S and 18S rDNA were amplified with Q5^®^ Hot Start High-Fidelity 2X Master Mix (NEB, M0494) and purified with QIAquick^®^ PCR Purification Kit (Qiagen, 28106) while 28S rDNA was amplified with KOD XtremeTM Hot Start DNA Polymerase (Millipore Sigma, 71975) and gel purified with QIAquick Gel Extraction Kit (Qiagen, and 28706X4). 100ng of purified DNA was used as a template for in vitro transcription using MEGAscript^™^ T7 Transcription Kit (Invitrogen, AM1334). Following incubation at 37C for 12 hours, in vitro transcription reactions were treated with TURBO DNase (ThermoFisher, AM2238) at 37C for 15 minutes to digest the DNA template and the IVT RNA was subsequently purified with the RNA Clean & Concentrator-25 (Zymo, R1018). Purified RNA concentrations were quantified using Nanodrop and verified for purity (single band) and size using an E-Gel EX Agarose Gel, 1% (ThermoFisher, G401001). All aforementioned kits were used according to their respective manufacturers’ instructions.

#### Microrheology of *in vitro* NPM1/rRNA droplets

Microrheology was performed in two-component condensates with final concentrations 10–20 μM NPM1 and 100 μg/mL rRNA (various species). Specifically, (i) purified total E. coli rRNA, (ii) in vitro transcribed (IVT) 18S rRNA, (iii) IVT 28S rRNA, or (iv) an equimolar mixture of 5.8S, 18S, and 28S IVT rRNA were used. Two separate solutions were prepared in 10 mM Tris, 150 mM NaCl, 2 mM DTT, pH 7.5. The first was an rRNA solution at double the desired final concentration (2x) that contained R=50 nm Red FluoSpheres^™^ Carboxylate-Modified Microspheres (ThermoFisher F8801). The second solution was a NPM1 purified protein solution at twice the desired final concentration (2x). These two solutions were combined equal volume (1:1) and mixed vigorously, added into an uncoated Ibidi μ-Slide Angionesis slides (81506), and covered with mineral oil to prevent evaporation. Droplets were allowed to settle for 20 minutes and 5 min time-lapse movies were acquired 1–2 μm above the coverslip with a 50 ms interval. Movies were analyzed using particle-tracking algorithms as described in detail in section “MSD tracking” above.

#### Purification and labeling of NPM1

The poly-His tagged wild-type NPM1 and a C21T/C275T mutant for fluorescence labeling were expressed in BL21 (DE3) E. coli cells (Millipore Sigma) and lysed in 25 mM Tris, 300 mM NaCl, 5 mM BME using sonication. After centrifugal clarification of the lysate, proteins were isolated from the soluble fraction using Ni-NTA affinity chromatography. The poly-His tag for both constructs was removed through proteolytic cleavage using TEV and further purified using HPLC with a C4 column (Higgins Analytical). Fractions containing the protein of interest were pooled and lyophilized. Lyophilized protein was resuspended in 20 mM Tris, 6 M GuHCl, pH 7.5 and refolded overnight using dialysis into 10 mM Tris, 150 mM NaCl, 2 mM DTT, pH 7.5. Refolding was confirmed using DLS and CD.

C21T/C275T NPM1 was fluorescently labeled with Alexa 594 dye (Thermo-Fisher) using maleimide chemistry according to the manufacturer’s protocol. Excess Alexa 594 dye was removed using HPLC with a C4 column (Higgins Analytical). Fractions of interest were pooled and lyophilized. Lyophilized NPM1-Alexa 594 was resuspended in 20 mM Tris, 6 M GuHCl, pH 7.5 and diluted 10-fold with unlabeled NPM1 to favor the incorporation of one labeled monomer per pentamer during refolding. The mixture was refolded using the procedure described above.

#### Purification of *E. coli* ribosomal RNA

E. coli K12, strain A19 cultures were grown at 37o in Lauria Broth to an OD600 of ~0.8 and harvested by centrifugation. Cell pellets were suspended in 20 mM Tris-HCl, 50 mM Mg(CH3CO2O)2, 100 mM NH4Cl, 1 mM TCEP, 0.5 mM EDTA, pH 7.5 and lysed using B-Per (Thermo-Fisher). The soluble fraction was layered onto a 30% sucrose cushion prepared in buffer A [10 mM Tris-HCl, 6 mM MgOAc, 50 mM NH4Cl, 1 mM TCEP, 0.5 mM EDTA, pH 7.5] and centrifuged at 30,000 rpm for 16 hours at 4° C in a SW 32 Ti Swinging-Bucket Rotor (Beckman Coulter). The resulting pellet was gently suspended in buffer A and subjected to two additional rounds of cushioning. Post cushioning, the suspended sample was purified using a 10–35% linear sucrose gradient prepared in buffer A and centrifuged at 32,000 rpm for 3 hours at 4° C and fractionated using a Piston Gradient Fractionator (BioComp). Samples containing pure 70S ribosomes were pooled and centrifuged at 30,000 rpm for 16 hours at 4° C. Resulting 70S ribosome pellet was resuspended in buffer A and the ribosomal RNA (rRNA) fraction (30S, 16S, and 5S; termed “total rRNA”) was extracted using phenol:chloform extraction. The RNA pellet was then suspended in 10 mM Tris, 150 mM NaCl, 2 mM DTT, pH 7.5 or stored at −20° C in 70% EtOH. RNA purity was confirmed through analysis using a 1.2% 1X Tris Borate EDTA denaturing agarose gel.

### QUANTIFICATION AND STATISTICAL ANALYSIS

#### Sphericity analysis and model

Cells expressing NPM1-mCherry and RPA16-GFP were imaged in three dimensions with z-stacks with a spacing of 0.3microns on a spinning-disk (Yokogawa CSU-X1) confocal microscope with a 100x oil immersion Apo TIRF objective (NA 1.49) and an Andor DU-897 EMCCD camera on a Nikon Eclipse Ti body. A 488 nm laser was used for imaging GFP and global activation, and a 561 nm laser for imaging mCherry. The imaging chamber was maintained at 37 °C and 5% CO2 (Okolab) with a 96-well plate adaptor.

Images are segmented and nucleoli are parsed as described below in the RDF calculation and analysis. Briefly, cells are manually segmented by polygon tracing and extraction, followed by automatic identification of the dense phase (“nucleolus”) as the highest intensity pixel and value after a slight blur and manual identification of a reasonable pixel and intensity to represent the dilute phase (“nucleoplasm”), and segmentation of nucleoli by utilizing these values to form a mask of the NPM1 channel. Then the identification of the true nucleolus surface and FC centers are also determined as described in the RDF analysis section below. To account for the additional blur in z for 3D data and to avoid anisotropic segmentation of the nucleolus, a slight erosion in z of 1 radial box is applied. To aid identification of the FC centers, a 2 pixel blur is applied in the x and y directions only. The nucleolar mask is then given to the function “BoundaryMesh” in Mathematica using the method DualMarchingCubes. This surface corresponds to that shown as “GC” in Figures 3B and S2B.

To produce the model image, we draw from the logic of transport theory in spherical coordinates where the concentration and flux is proportional to 1/r and 1/r^2^, respectively and r is the distance from a source (which in this case is the FC center). At steady state, the flux of radiating material is balanced by a sub-saturation. To roughly incorporate these concepts into a model, we calculate the total flux at each position as a sum of 1/r^2^ over all FC centers using the same grid and spacing as the image. Then, the cut-off value for the surface (where all voxels greater than the total flux of the cut-off value are included as part of the nucleolus surface) is chosen such that the model will have the same number of voxels as the data. The boundary mesh is then produced as done with the data nucleolar mask. This surface corresponds to that shown as “Model” in Figures 3B and S2B.

To approximate the degree of agreement between the two surfaces, spherical harmonics are calculated for the surface using the pseudo-inverse of the linear system with regularisation as in.^83^ Then the Pearson correlation coefficient is calculated. Note that for complicated nucleolar surfaces this analysis is not robust to reproduce the surface due to the breakdown of the 1:1 relationship between the polar and azimuth angles and the radial distance. The statistics quoted and Figure S2C are shown for HEK data only.

#### RDF calculation and analysis

The formula for the radial distribution function (RDF) utilized throughout the manuscript is: $\frac{<I\left(r+\Delta r\right)M\left(r+\Delta r\right)\delta \left(r\right)>}{<I\left(r\right)M\left(r\right)><M\left(r+\Delta r\right)\delta \left(r\right)>}$; where I(r), M(r), and δ(r) are the background-subtracted image intensities (e.g. NPM1 signal), the binary mask for the region of interest (here the nucleolus), and a mask marking the pixel locations of the radial centers (here the center of the FC) at a given location r, respectively.

To calculate RDFs, confocal images of fixed cells in one z plane are taken as described above. To avoid bias, cells are first manually segmented via chosen polygon outlines blinded to the EU channel (or FIB channel for non-EU RDF experiments). Background subtraction is then performed followed by segmentation of each cell using the polygonal regions identified. The cell segmented images are then used to identify the location and intensity of the dense phase (e.g. concentration of NPM1 in the GC phase of the nucleolus) by programmatically reporting the brightest pixel location and intensity after a 2 pixel radius blur of the image to remove noise. Then looking at the NPM1 image for each cell after a 5 pixel radius blur, log transformed, and normalized to remove bias on the extent of NPM1 overexpression, a reasonable representative location of the nucleoplasm is identified and the intensity (only after the 5 pixel radius blur) is designated the dilute phase concentration for each channel. At this point, the NPM1 channel is used for each cell to form a mask by binary assignment to pixels greater than 0.25 the intensity value between the dilute and dense phase intensities followed by a filling transform, a deletion of small components with less than 300 contiguous pixels, and morphological segmentation (“MorphologicalComponents” in Mathematica) ignoring corner pixels. From these, any components less than 9 pixels large are discarded. The rest are designated as nucleoli.

For each nucleolus, the centers of FCs are identified using a combination of thresholding, distance transform, max detection, and watershedding. The nucleolus mask is then filled and eroded with a 3 pixel square to remove the expected lower intensity at the surface due to resolution limits; this mask is applied to each channel and FC centers not within this mask are discarded. Finally, to calculate the RDF, the image correlation between each channel with the image of the FC centers weighted by their relative intensity (blurred over one pixel radius for slight integration) is divided by the average intensity of the image channel. The denominator of the RDF is the image correlation between the nucleolar mask and the weighted FC centers which corresponds to the number of positions within the nucleolus at a specific distance from any FC center. To average the nucleoli, numerator and denominator RDF values at all displacements are calculated for each nucleolus and those that are smaller than the desired range of displacements are discarded; then to calculate the RDF value the the total numerator is divided by the total denominator of all remaining nucleoli. To approximate the error on the RDF value, error propagation is utilized by taking advantage of the fact that the denominator RDF corresponds to the weighted number of pixels which are being probed at a specific displacement value.

Throughout the text unless otherwise indicated the RDF points are shown with a spline fit to better depict the RDF trends and error. This is done in Mathematica by fitting a linear model with a Bernstein Basis where the input is normalized by the largest dimension in the RDF fit and raised to the 1.8 power to account for the non-linear spacing in displacement values between 0 and 1μm shown throughout the text. The number of splines utilized is 7 for the data fit between the displacements of 0 and 1μm shown primarily throughout the text. This is adjusted based on the 1.8 scaling in the displacement values and corresponds to 15 for the largest interval (2.5μm displacement) and 10 for the intermediate one (1.5μm displacement) used in the concentration dependence to account for the growing nucleolus with higher NPM1 overexpression as discussed below.

To determine concentration dependence, we fit the RDF dependence on the dense phase concentration of the NPM1 signal linearly (Figures S6B and S6C). The shown dependence on $\Delta G_{NPM1}^{tr}$ is determined as done previously^15^ with the addition offset to account for additional background in stained cells autofluorescence that is noticeable in the trend (Figure S6A). At each displacement, the RDF is extrapolated to the average, minus one standard deviation, plus one standard deviation, and plus two standard deviations in the dense phase NPM1 concentration. As the $\Delta G_{NPM1}^{tr}$ is dimensionless, we convert these dense phase concentrations into these in the text.

#### Spherical diffusion-advection equation and application

To determine the flux of RNA throughout the nucleolus we utilize the incompressible advection-diffusion equation (Equation 1) where D is the diffusion constant and v is the velocity.


(Equation 1) $$\partial_{t}c=\nabla \cdot (D\nabla c-vc)$$

Because the radial distribution function (RDF) is spherically averaged, we can reduce the general equation to the case for spherical flow (Equation 2) dependent only on the radial distance, r, and where the flow velocity (v) is dependent on the injection rate (Q), through the relation $v=\frac{Q}{4\pi r^{2}}$. This assumes incompressibility of the RNA.


(Equation 2) $$\frac{\partial c}{\partial t}=D\left(\frac{2}{r}\frac{\partial c}{\partial r}+\frac{\partial^{2}c}{\partial r^{2}}\right)-\frac{Q}{4\pi r^{2}}\frac{\partial c}{\partial r}$$

Simplifying to dimensionless units with $\rho =\frac{r}{R},\;Pe=\frac{Q}{4\pi DR}$, and $\tau =\frac{Dt}{R^{2}}$, where τ is the dimensionless time, ρ is dimensionless radius, Pe is the dimensionless Peclet number describing the ratio between advection and diffusion, and R is the outer boundary, yields Equation 3.


(Equation 3) $$\frac{\partial c}{\partial \tau }=\left(\frac{2}{\rho }\frac{\partial c}{\partial \rho }+\frac{\partial^{2}c}{\partial \rho^{2}}\right)-\frac{Pe}{\rho^{2}}\frac{\partial c}{\partial \rho }$$

To solve Equation 3 we use separation by parts method, also known as the fourier method.^84^ In the separation by parts method, basis solutions, $\beta_{i}(\rho ,\tau )$, to this equation are solved which obey the relationship $\beta_{i}(\rho ,\tau )=\chi_{i}(\rho )\zeta_{i}(\tau )$. To determine the solutions, we start by defining the eigenvalues, $\lambda_{i}$, of the basis solutions as Equation 4A which simplifies to Equation 4B.


(Equation 4A) $$\lambda_{i}=-\frac{1}{\beta_{i}(\rho ,\;\tau )}\frac{\partial \beta_{i}(\rho ,\;\tau )}{\partial \tau }$$

(Equation 4B) $$-\lambda_{i}\beta_{i}(\rho ,\tau )=\frac{\partial \beta_{i}(\rho ,\;\tau )}{\partial \tau }$$

By plugging in the relationship for $\beta_{i}$ and Equations 3 and 4 we obtain Equations 5 and 6.


(Equation 5) $$-\lambda_{i}\zeta_{i}(\tau )=\frac{\partial \zeta_{i}(\tau )}{\partial \tau }$$

(Equation 6) $$-\lambda_{i}\chi_{i}(\rho )=\frac{2}{\rho }\frac{\partial \chi_{i}(\rho )}{\partial \rho }+\frac{\partial^{2}\chi_{i}(\rho )}{\partial \rho^{2}}-\frac{Pe}{\rho^{2}}\frac{\partial \chi_{i}(\rho )}{\partial \rho }$$

Equation 5 can easily be solved to Equation 7.


(Equation 7) $$\zeta_{i}(\tau )=\zeta_{i}(0)e^{-\lambda_{i}\tau }$$

While there exists no simple form using standard commonly defined functions for the solution to Equation 6, these can be solved numerically in mathematica with the function “NDEigensolution” which yields both $-\lambda_{i}$ and the numerical solution for $\chi_{i}(\rho )$, henceforth called the eigenfunction, for the *i*th largest eigenvalue. We use a Dirichlet boundary condition equal to zero at the center and Neumann boundary condition of zero at the outer boundary (i.e. R) allowing for the constant expulsion of material due to the nature of the pulse chase experiment.

To determine the apparent Pe inside the GC phase of the nucleolus, only the smallest (i.e. index ‘zero’) eigenvalue’s eigenfunction $\chi_{0}(\rho )$, or $\chi_{0}(Pe,\rho )$ due to its dependence on the Peclet number, is needed; this is due to the fact that the RDF is an intensity/concentration normalized profile and thus corresponds to the apparent steady state of the RDF curve. To apply this to data, we fit the RDF for the EU (i.e. $RDF_{EU}(\rho )$) data using $\chi_{i}(Pe,\rho )$, the EU signal of NPM1 (i.e. ${RDF}_{NPM1}(\rho )$), and three ad-hoc parameters (α,β,γ), that are intended to account for experimental realities such as the diffraction limit) with Equation 8.


(Equation 8) $$RDF_{EU}(\rho )==\alpha^{2}+\left(RDF_{NPM1}(\rho )-\alpha^{2}\right)\left(\beta^{2}\chi_{0}(Pe,\rho )+\gamma^{2}\right)$$

To fit the full dataset, we use R=1μm to make the displacement values into the dimensionless ρ. In the concentration dependent series, we adjust R by the fold increase in the maximum peak displacement in the RDF of NPM1 relative to that at no overexpression. To determine the kinetics of the RDF profile, we solve for the 20 highest eigenfunctions and eigenvalues. Using these eigenfunctions, we solve for a linear combination of them with prefactor $\zeta_{i}(\tau )$ that yields a peaked solution near the origin at τ=0 and produces no significant negative value concentration profiles at all times. To convert this into an RDF we normalize the solution by the average value from the center to the outer boundary of the sphere. When showing the model in Figure 3C, we use unit diffusion and either a Pe of 0 or 1 for the diffusion only and diffusion plus advection cases. Furthermore in this case we use the starting conditions shown. To fit the time dependence, we fit the RDF data at ρ~0.9 (averaging the RDF values between the dimensionless displacements of 0.8–1) fitting for the diffusion constant D, a time offset, and baseline and scaling factors. The Pe number is set to 2 being the best fit to the full dataset. The outer radius, R, is set as described above.

#### Half-FRAP and FRAP diffusion analysis

To determine the diffusion of nucleolar proteins following half-FRAP as described above, we will use the separation by parts as described in the previous section. Unlike the spherical transport solution, half-FRAP can be approximated as a 1D diffusion where advection can be ignored due to the fast recovery of proteins in the nucleolus. Thus we begin with the simple 1D linear diffusion equation:

$$\frac{\partial c}{\partial t}=D\left(\frac{\partial^{2}c}{\partial x^{2}}\right)$$

Where D is the diffusion constant. To dimensionalize the solution, we substitute $\xi =\frac{x}{L}$ and $\tau =\frac{Dt}{L^{2}}$ where L is the length of the nucleolus yielding:

$$\frac{\partial c}{\partial \tau }=\frac{\partial^{2}c}{\partial \xi^{2}}$$

The solution to this equation is a wave (e.g. Sinusoidal). Now solving this equation from ξ between negative and positive half using the separation by parts yields the solution:

$$c(\tau ,\xi )=1+\sum_{i=-\infty }^{\infty }\;\frac{cos\left(n\frac{\pi }{2}\right)-1}{n\pi }sin(n\pi \xi )e^{-(n\pi {)}^{2}\tau }$$

This equation is fit to half-FRAP data at ξ=14 corresponding to the location in the middle of the bleached half. Thus we fit the data using NonlinearModelFit in Mathematica to:

$$Ac\left(\frac{Dt}{L^{2}},\frac{1}{4}\right)$$

Where D,A, and L are the diffusion constant, fraction intensity recovered, and the average size of the nucleoli fit, being 4 microns. c is approximated by interpolation of the aforementioned solution with the summation truncated to between −40 and 40 using NonlinearModelFit in Mathematica.

To fit the corelet FRAP data, we fit a stretched exponential decay, $A\left(1-2^{-{\left(\frac{t}{\tau }\right)}^{\lambda }}\right)$, using NonlinearModelFit.

#### MSD tracking

To track corelet droplets, images were taken every 3 seconds for 100 minutes following 5 minutes of initial activation on the aforementioned spinning disk as described in ^37^. For FC tracking NPM1-mcherry and RPA16-GFP (RPA16-miRFP in case of HP1 and LMNA knockdown) were taken approximately every 20 seconds for 2.5 hours.

For all datasets, subpixel tracking was performed in TrackMate^85^ using a Laplacian of Gaussians filter-based detector and a blob diameter of 500 nm (or an appropriate size for local activation experiments with large droplets), a threshold of 250. For tracking of FCs in the HP1 and LMNA knockdown experiments, thresholds of 65–75 (adjusted to suit individual samples) and a diameter of 600nm was used. For tracking of probes in reconstituted GCs, threshold of 20 and diameter of 800nm were used. Probes residing in the periphery (defined as 10 times the bead diameter away from droplet edge) for more than 20% of the track were excluded from analysis. Trajectories were then constructed using the simple linear assignment problem (LAP) tracking with max linking and gap-closing distances of 500 nm and no frame gap accepted. Coordinates parsed into MATLAB and pair MSDs were calculated with custom routines. In mathematica, we fit the Maxwell model equation, $<\Delta r^{2}\geq \Delta_{0}^{2}\left(1+\frac{t}{\tau }\right)$, to the data.

#### Fusion

To visualize corelet fusion events, large droplets were generated by local activation using a Mightex Polygon digital micromirror device (485nm) and imaged using a 561nm laser at 3 seconds per frame on the aforementioned spinning disk. To visualize nucleolar merger, images of NPM1-mCherry and RPA16-GFP were taken approximately every 20 seconds for 2.5 hours. Images of merging condensates were analyzed in Fiji and MATLAB; the aspect ratio was calculated by taking the ratio of the major and minor axes (‘*regionprops*’) at each timepoint.

#### Overlap concentration

The concentration where polymers begin to become entangled is referred to as the overlap concentration.^51^ The overlap concentration (in units of the number of chain particles per volume) is approximated as $C^{*}\;\sim \frac{1}{R^{3}}$ where R is the average (RMS) end-to-end distance of the chain.^51^ Given the measured end-to-end distance of the 16S (prokaryotic) rRNA (~1.5kb) of ~50 nm,^52^ the ratio of the number of basepairs or monomers between the 47S rRNA (~13.3kb) and the 16S rRNA is ~9, and the assumption that the chain acts roughly as a random walk where the end to end dimensions scale as the square root of the number of monomers, the end-to-end distance R of the full rRNA transcript is ~150 nm. Calculating the overlap concentration for this end-to-end distance yields ~300 molecules per μm or ~0.5 μM. On the other hand, full folded ribosomal subunits are anticipated to have an end-to-end distance of ~10^3^ nm giving them an overlap concentration of ~10^6^ molecules per μm^3^ or ~1700μM. Thus the calculated overlap concentration increases over 3,000 fold during the folding of the nascent rRNA into ribosomal subunits; in other words, rRNA becomes about 3,000 fold less entangled as it goes from the nascent chain to the folded subunit.

To determine how the actual rRNA concentration compares with the overlap concentrations for both nascent and mature transcripts, we utilize curated values for the transcription $rate(k_{trans}\;\sim 10^{3.5}\;transcripts/min)$, rRNA residence time $\left(\tau_{rRNA}\;\sim 45\;min\right)$, average nucleolar radius $\left(R_{nucl}\;\sim 1.5um\right)$, and average number of nucleoli $\left(N_{nucl}\;\sim 3.5\right)$.^86^ From the relationship $C_{rRNA}=\frac{k_{trans}\tau_{rRNA}}{\left(N_{Nucl}\right)\left(\frac{4}{3}\pi R_{nucl}^{3}\right)}$, these data imply that roughly 1.5*10^5^ rRNA molecules dwell within the total average nucleolar volume of 50 μm^3^, yielding a concentration ~3000 molecules per μm^3^ or ~5μM. Since this is higher than the overlap concentration for nascent rRNA chains, i.e. 5μM>0.5μM, this implies that they are significantly entangled, as can be visualized in Figure 5A. On the other hand, since the rRNA concentration is much less than the overlap concentration for folded rRNA subunits, i.e. and 5μM<<2,000μM, assembled ribosomal subunits are certainly not entangled. The nascent 13.3kb rRNA transcript is cleaved into several fragments, including the 5.8S (length=160bp), 28S (length=4.7kb), and 18S (length=1.9kb), which can partially help disentangle the chains. However, since the overlap concentration scales with the inverse physical size of the chain R (i.e. ${R_{ee}}^{3}$ or ${R_{g}}^{3}$), and making the simplifying approximation that the chains behave as random coils yields a dependence of $R\;\sim N^{2}$, there is a relatively weak dependence of the overlap concentration on the number of nucleotides comprising the chain, i.e. $C^{*}\;\sim N^{-2}/3$. As a consequence, folding of the chain, i.e. going from a random-coil to a more compact structure dictated by its intra- and inter-molecular interactions, is likely the more important contribution to the progressive loss of rRNA entanglement.

## Supplementary Material

1

## Figures and Tables

**KEY RESOURCES TABLE:** 

REAGENT or RESOURCE	SOURCE	IDENTIFIER

Antibodies		

Rabbit polyclonal anti-fibrillarin	Abcam	Cat#:5821;RRID:AB_2105785
AlexaFluor647 goat-anti-rabbit	Thermo Fisher	Cat#:A-23245
Mouse anti-NPM1 AlexaFluor647	Thermo Fisher	Cat#:MA3-25200-A647;RRID:AB_2663611
Anti-LaminA/C	Active Motif	Cat#:36287
Goat anti-mouse Alexa488	Thermo Fisher	Cat#:A-11029

Bacterial and virus strains		

BL21 (DE3) E.coli	Millipore Sigma	Cat#:69450
K12 A19 E.Coli	Kriwacki Lab, St Jude	N/A

Chemicals, peptides, and recombinant proteins		

Fetal Bovine Serum, Premium, Heat-Inactivated	Atlanta Biologicals	Cat#:S11150H
GIBCO DMEM, High Glucose	Thermo Fisher Scientific	Cat#:11-965-118
GIBCO Penicillin-Streptomycin (10,000 U/mL)	Thermo Fisher Scientific	Cat#:15140122
Trypsin-EDTA 0.05%	Fisher Scientific	Cat#:25300054
Fibronectin bovine plasma	Millipore Sigma	Cat#:F1141
Lipofectamine 3000 Transfection Reagent	Invitrogen	Cat#:E2311
In-Fusion HD-Cloning	Takara Bio USA	Cat#:638910
Papain dissociation system	Worthington Biochemical Corp	Cat#:LK003160
Gibco B-27^™^ Plus Supplement (50X)	Thermofisher	Cat#:A3582801
GIBCO Neurobasal plus medium	Thermofisher	Cat#:A3582901
GIBCO CultureOne supplement	Thermofisher	Cat#:A3320201
Poly-D-Lysine	Thermofisher	Cat#:A3890401
Matrigel	Corning	Cat#:354277
mTeSR Plus medium	Stem Cell Technologies	Cat#:100-0276
ROCK inhibitor	Selleckchem	Cat#:Y-27632
GIBCO 30% Knock out serum replacement	Life Technologies	Cat#:10828-028
Lenti-X concentrator	Takara Bio USA	Cat#:631231
Click-iT RNA imaging kit	Thermo Fisher Scientific	Cat#:C10330
AlexaFluor647 Azide Triethylammonium Salt	Thermo Fisher Scientifc	Cat#:A10277
ActinomysinD	Sigma	Cat#:A5156-1VL
CX-5461	Selleckchem	Cat#:S2684
Auxin	Sigma	Cat#:I5148-2G
Hoechst	Thermo Fisher Scientific	Cat#:62249
Lipofectamine RNAiMAX	Invitrogen	Cat#:13778075

Experimental models: Cell lines		

Human: HEK293	Marc Diamond, UTSW	N/A
Human: Lenti-X 293T	Takara Bio USA	Cat#:632180
Human: U2OS	Tom Muir, Princeton	N/A
Human: iPSC	Allen Institute Cell Science	Cat#:AICS-0084-018
Monkey: Vero	ATCC	Cat#:CCL-81
HEK293+NPM1-mCh+RPA16-GFP	This paper	N/A
HEK293+FusN-mCh-sspB+pHR-SFFVp-NLS-iLID-eGFP-FTH1	This paper	N/A
Human: NIH-3T3-mCh-Cry2Olig	Zhu et al. ^56^	N/A
U2OS+HP1-AID	Strom et al. ^40^	N/A

Experimental models: Organisms/strains		

Timed-pregnant Sprague-Dawley rats	Hilltop Lab Animals	Hla:(SD)CVF

Oligonucleotides		

5.8S FW (In vitro transcription of rRNA species)	GGATTCTAATACGACTCACTATAGG GGACTCTTAGCGGTGGATCACTCGG	IDT
5.8S Rev (In vitro transcription of rRNA species)	AAGCGACGCTCAGACAGGCG	IDT
18S FW (In vitro transcription of rRNA species)	GGATTCTAATACGACTCACTATAGGGA CCTGGTTGATCCTGCCAGTAGCATATG	IDT
18S Rev (In vitro transcription of rRNA species)	TAATGATCCTTCCGC AGGTTCACCTACGGAA	IDT
28S FW (In vitro transcription of rRNA species)	GGATTCTAATACGACTCACTATAG GGGCGACCTCAGATCAGACGTGGC	IDT
28S Rev (In vitro transcription of rRNA species)	GACAAACCCTTGTGTCGAGGGC	IDT
anti-Lamin A RNAi construct	Ambion	Cat#:4427038
Cy5 labeled control siRNA	Signal Science	Cat#:86921
Alexa594 Maleimide	Thermo Fisher Scientific	Cat#A10256
tC nucleotide	Wang et al.^77^	N/A
FluoroBrite media	ThermoFisher scientific	Cat#:A1896702

Recombinant DNA		

RPA16	Zhu et al.^78^	N/A
RPL5	Gene block	IDT
FM5-stdlinker-mCh	Brangwynne lab	N/A
FM5-stdlinker-eGFP	Brangwynne lab	N/A
FM5-stdlinker-mCh-sspB	Brangwynne lab	N/A
pHR-SFFVp-NLS-iLID-eGFP-FTH1 (Daniel check)	Bracha et al.^8^	N/A
FM5-NPM1-mCh	Riback et al.^79^	N/A
FM5-RPL5-eGFP	This paper	N/A
FM5-RPA16-eGFP	This paper	N/A
pHR-FusN-mCh-sspB	Brache et al.^80^	N/A
Uck2 kinase	Wang et al.^77^	N/A

Software and algorithms		

FIJI (ImageJ 1.52p)	Schindelin et al. ^81^	http://fiji.sc
Matlab 2019b	Mathworks	https://www.mathworks.com/products/MATLAB.html
Mathematica	Wolfram	https://www.wolfram.com/mathematica/

## Highlights

- Nucleoli are complex fluids driven by rRNA transcription
- Chromatin constrains nucleoli and unexpectedly increases their sphericity
- Advective transport plays a significant role in rRNA motion
- Results suggest entanglement and viscoelasticity facilitate ribosome maturation
