# Supplementary material for: Viscoelasticity and advective flow of RNA underlies nucleolar form and function
Source: Mol Cell. Author manuscript; Available in PMC 2024 May 13. (PMC11089468; doi:10.1016/j.molcel.2023.08.006)
Supplement: 1 [file NIHMS1941406-supplement-1.pdf]

Molecular Cell, Volume *83*

Supplemental information

Viscoelasticity and advective flow of RNA

underlies nucleolar form and function

Joshua A. Riback, Jorine M. Eeftens, Daniel S.W. Lee, Sofia A. Quinodoz, Anita Donlic, Natalia Orlovsky, Lennard Wiesner, Lien Beckers, Lindsay A. Becker, Amy R. Strom, Ushnish Rana, Michele Tolbert, Byron W. Purse, Ralph Kleiner, Richard Kriwacki, and Clifford P. Brangwynne

Supplementary Figures:

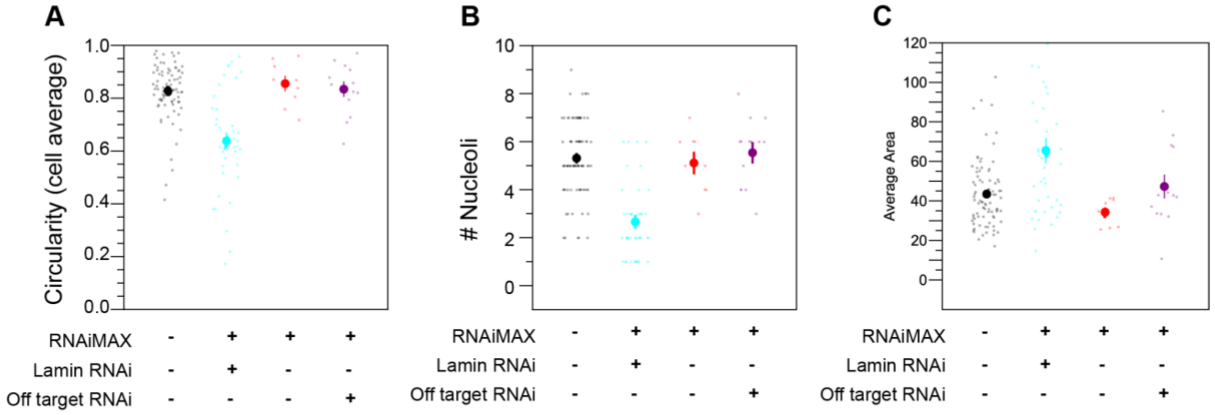

Figure S1: Knock-down of Lamin A leads to nucleolar shape changes, related to Figure 2. A) Circularity of nucleoli in Lamin KD cells compared to controls. B) Number of nucleoli. C) Total area.

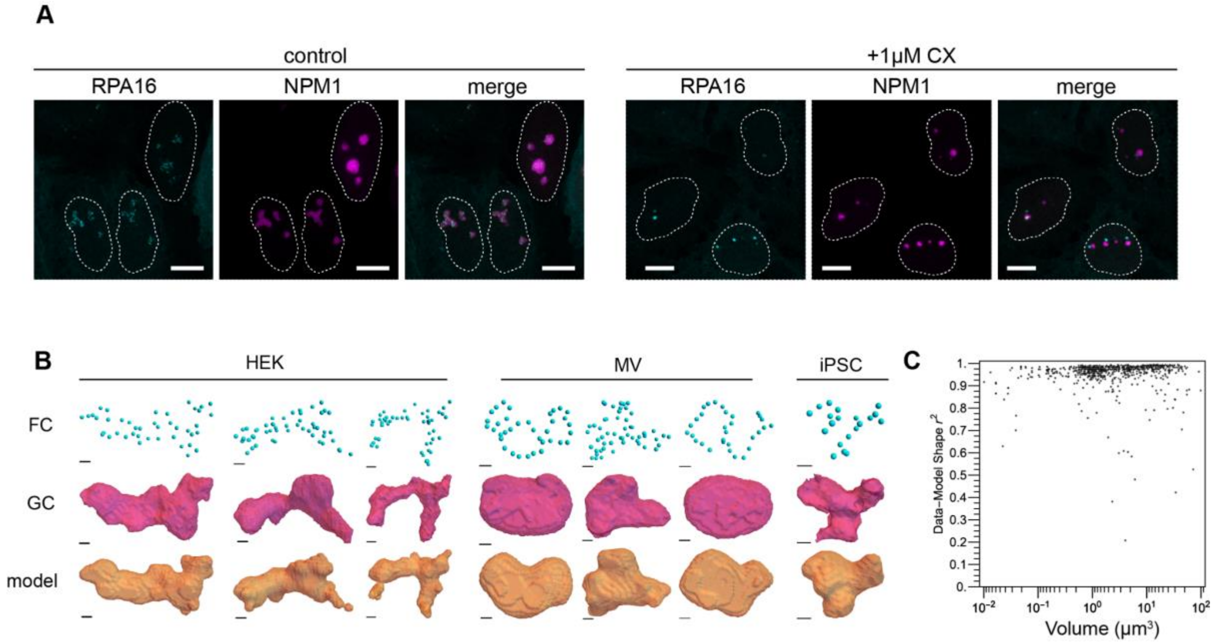

Figure S2: Additional examples for nucleolar shape analysis, related to Figure 3. A) Cells treated with Pol I inhibitor CX also show nucleolar capping. B) NOR location is sufficient to describe nucleolar shape, additional examples in multiple cell types from **Figure 3B**. C) Spherical harmonic decomposition shows strong agreement ($R^2 = 0.96 \pm 0.01$) between data (i.e., GC segmented surface) and model (i.e., FC derived nucleolar shape) with little dependence on the total volume of the nucleoli.

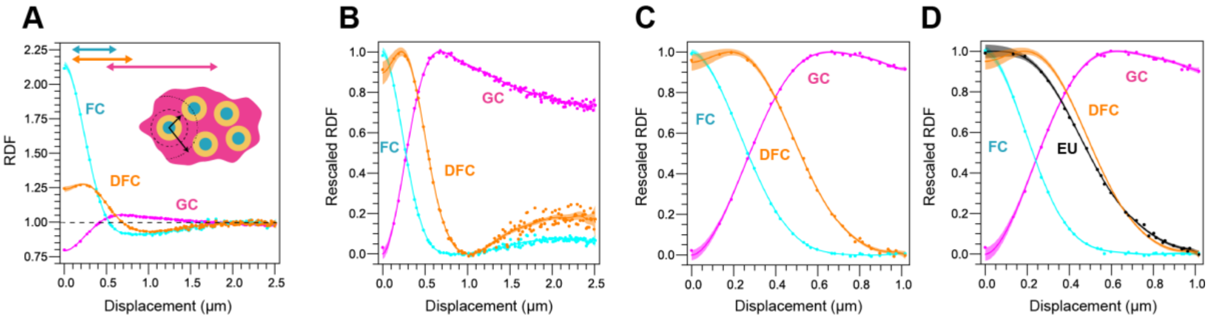

Figure S3: Stepwise RDF calculation, related to Figure 3. A) Same as **Figure 3D**, the RDF of nucleolar phases follows concentrically. B) Rescaled RDF, with the DFC curve exhibiting a minimum point at ~1µm, indicating where adjacent FCs start to contribute to the RDF. C) RDF focused on the relevant part <1µm (here, N=333 nucleoli; this is increased due to the inclusion of smaller nucleoli from the smaller displacement maximum, see methods). Unlike **Figure 3E**, here all data is from the same experiment. D. Same as **Figure 3E**, for comparison to **Figure S3C**.

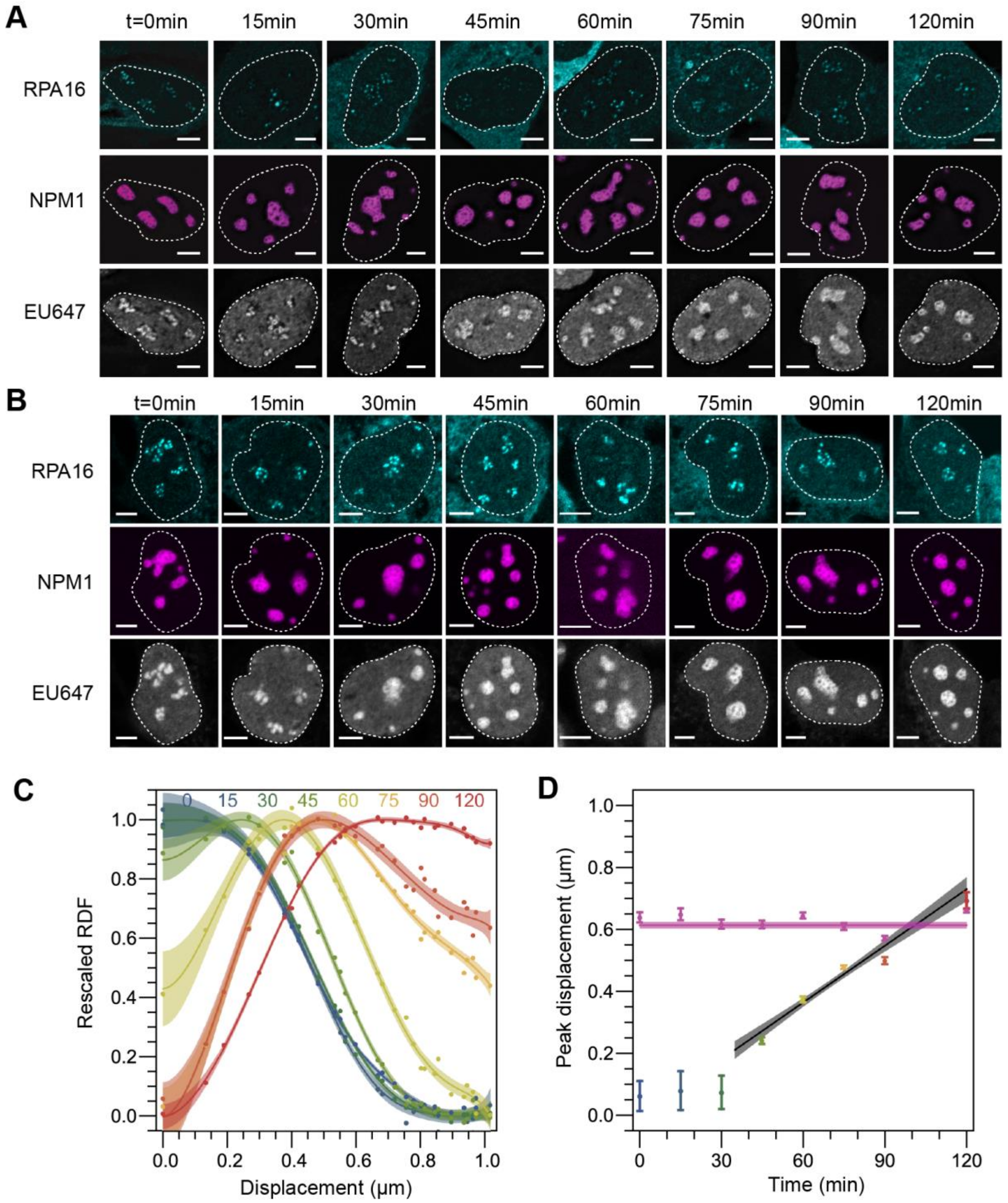

Figure S4: Replicates of pulse chase experiment, related to Figure 4. A) Nuclei showing EU dynamics over time. Nucleoli in Figure 4B are taken from this series. B) Replicate of experiment shown in A. C) Rescaled RDF quantification of EU signal from replicate shown in B (N= 60, 55, 52, 55, 92, 61, 66, and 77 nucleoli for sequential timepoints). D) RNA peak over time where linear fit is ~1Å/s as with **Figure 4E.**

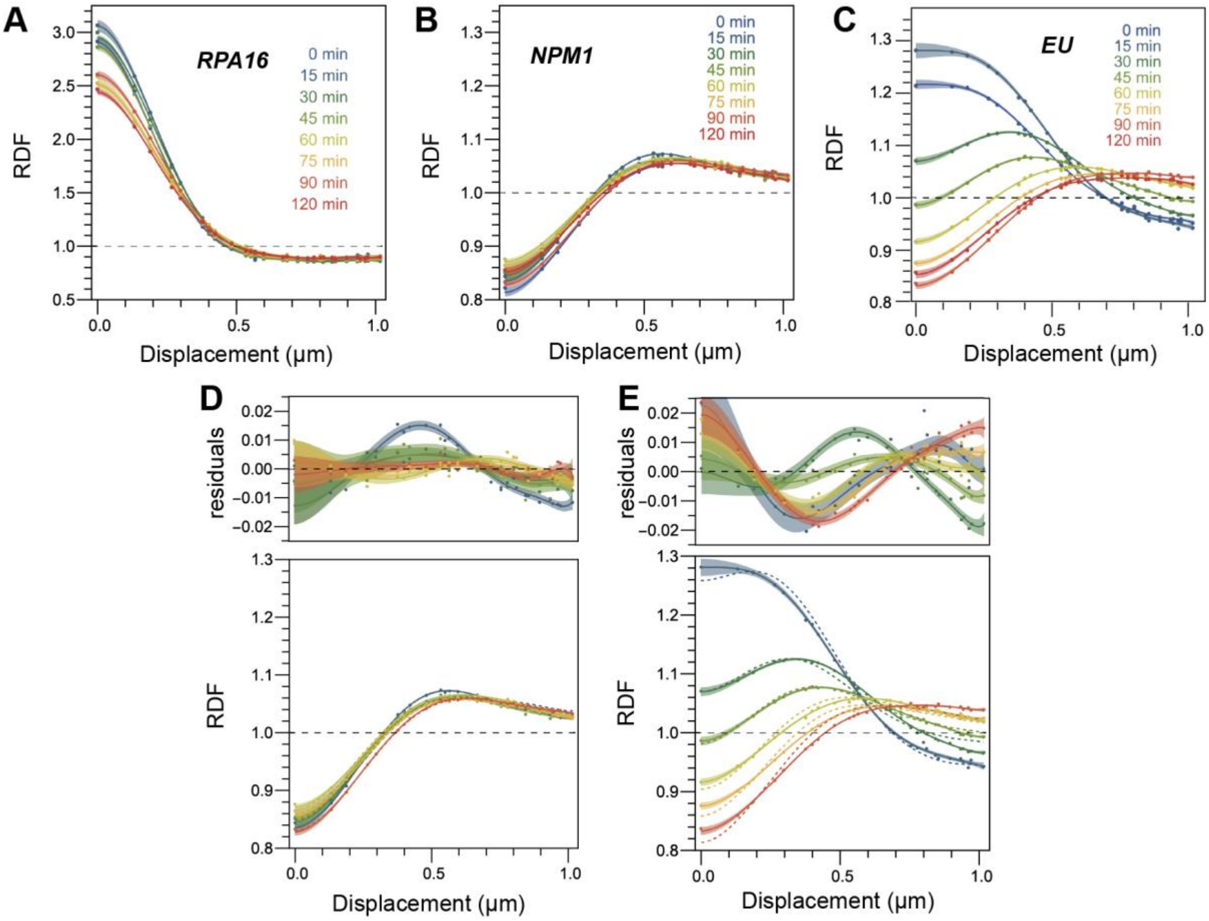

Figure S5: RDF validation, related to Figure 4. A) The RDF of RPA16 over time. B) The RDF of NPM1 over time. C) The RDF of EU over time (same as **Figure 4C**). D-E) Rapid-partitioning model between the DFC and GC (i.e. linear combination of basis RDFs for DFC and GC) reasonably fits the NPM1 data (E) but fails to fit the EU data (F). Top, residuals between data and model with error curves and fit lines using spline fit to data. Bottom, dashed lines show best fit from model compared to data and spline fit to data.

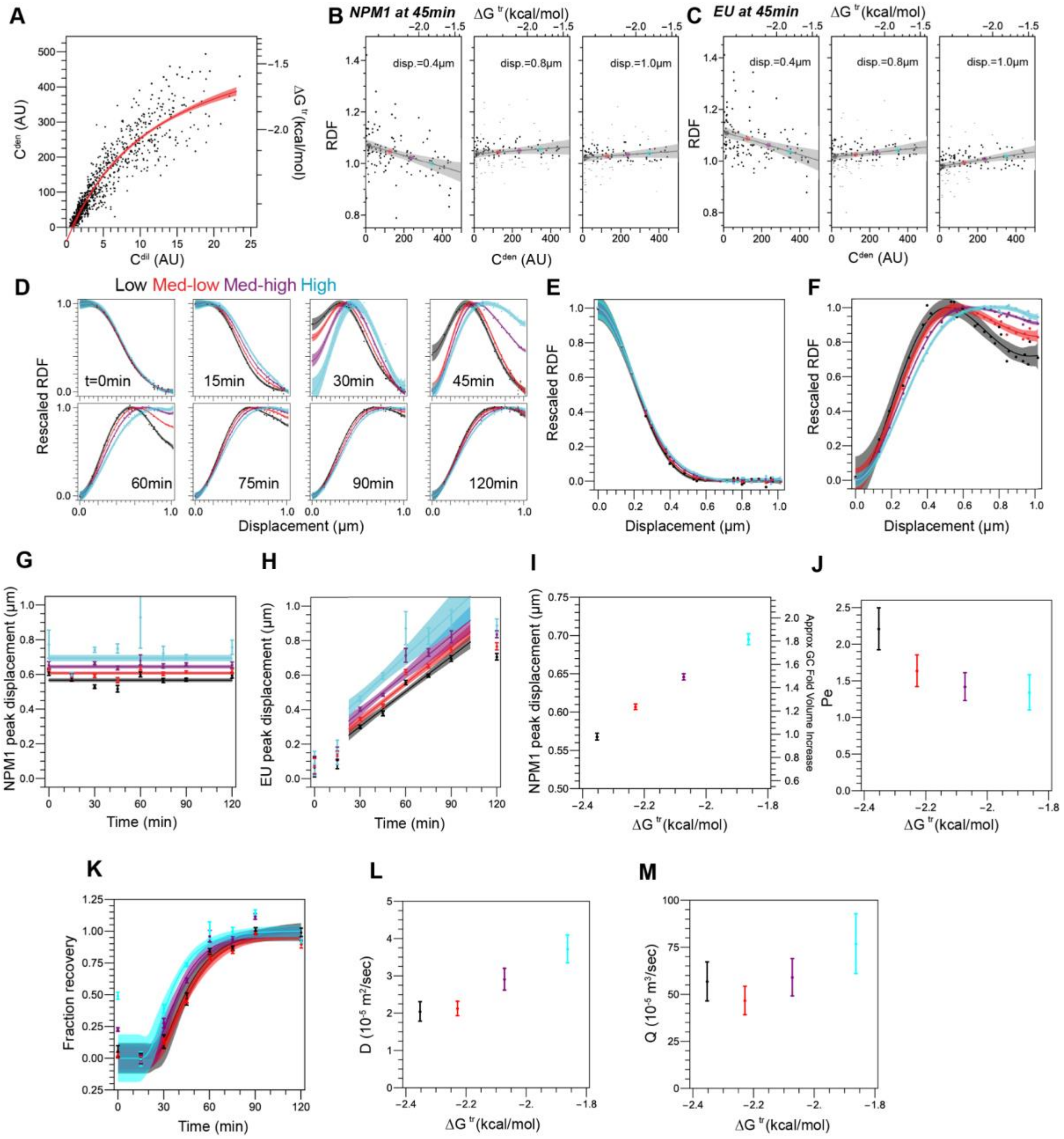

Figure S6: NPM1 concentration dependence on RDF curves, related to Figure 4. A) Dependence of NPM1 dense and dilute concentrations with NPM1 overexpression. Corresponding approximate

ΔG^{tr} shown on the right axis. B) RDF of NPM1 at t=45min at different displacements for different NPM1 concentrations. C) RDF of EU at t=45min at different displacements for different NPM concentrations. For B and C, dots correspond to concentration of NPM1 used for subsequent labels of the degree of NPM1 overexpression. D) Rescaled RDF of EU at different timepoints for different NPM1 concentrations. E-F) NPM1 concentration dependence of rescaled RPA16 (E) and NPM1 (F) RDF at 45min; these show the lack and significant dependence of FC and GC structure, respectively on NPM1 concentration. G) NPM1 peak displacement over time at different NPM1 concentrations. H) EU peak displacement over time at different NPM1 concentrations. I) NPM1 peak displacement from (G); note 25% increase in peak displacement from low to high over expression corresponds to roughly a doubling of GC volume (i.e., 1.25^3 =~2). J) Peclet number dependence on NPM1 concentrations. K) NPM1

concentration dependence on the EU movement to the edge of the GC. L-M) Diffusion constant (L) and advection (M) dependence on NPM1 concentration.

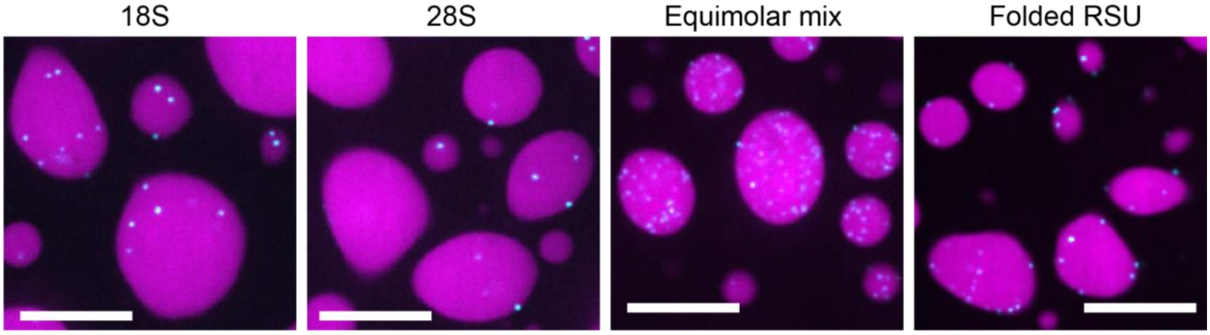

Figure S7: In vitro reconstitution of GC with RNA, related to Figure 5. Scale bar is 10µm.

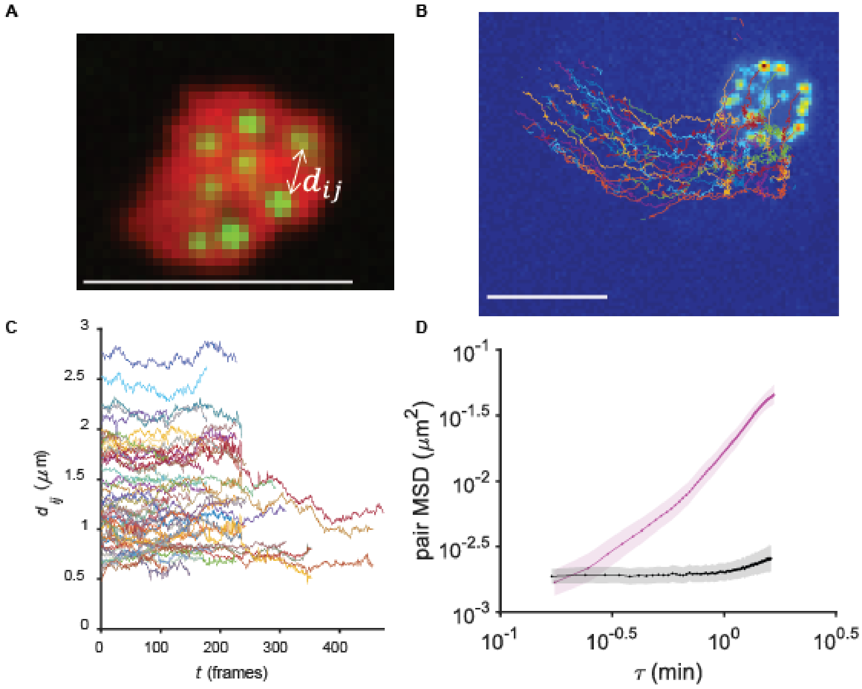

Figure S8: pair-MSD calculation details and controls, related to Figure 5. A) To characterize NOR dynamics, cells co-expressing RPA-GFP and NPM1-mCherry were imaged for 2.5 hours at an interval of 20 seconds per frame and individual nucleoli were analyzed as shown. Scale bar is 5µm. B) Single particle tracking of RPA foci revealed bulk motion associated with individual nucleoli and nuclei. C) In order to correct for this, following[1], the displacement was computed for each individual displacements (as shown in **Figure. S8A**) for each possible pair of foci. D) The pair MSD was calculated from pair displacements and averaged over all pairs within individual nucleoli and then over the population of nucleoli. Pair trajectories with fewer than 100 continuous frames and time lags of fewer than 90 frames were excluded from the analysis. Noise floor calculating for pair MSD was calculated by tracking pairs of RPA foci in fixed U2OS cells (531 pairs in 20 nucleoli), plotted in black. HEK pair MSD (508 pairs in 7 nucleoli) presented in main text plotted in magenta, demonstrating that the first two points are near noise floor but remaining lags are far above it, suggesting that NORs dynamics transition from a subdiffusive to a diffusive regime.

Supplemental References:

1. Khanna, N., Zhang, Y., Lucas, J.S., Dudko, O.K., and Murre, C. (2019). Chromosome dynamics near the sol-gel phase transition dictate the timing of remote genomic interactions. Nat. Commun. *10*, 2771.
